# Supplementary material for: The impact of the Syrian conflict on population well-being
Source: Nat Commun. 2020 Aug 6;11:3899. doi: 10.1038/s41467-020-17369-0 (PMC7411009; doi:10.1038/s41467-020-17369-0)
Supplement: Supplementary file 1 — Supplementary Information [file 41467_2020_17369_MOESM1_ESM.pdf]

**Supplementary Information**

# The Impact of the Syrian Conflict on Population Well-being

Cheung et al.

Corresponding author: Dr. Felix Cheung
Email: felixckc@hku.hk

**The supplementary file includes:**

Supplementary Figures 1 to 5
Supplementary Tables 1 to 15

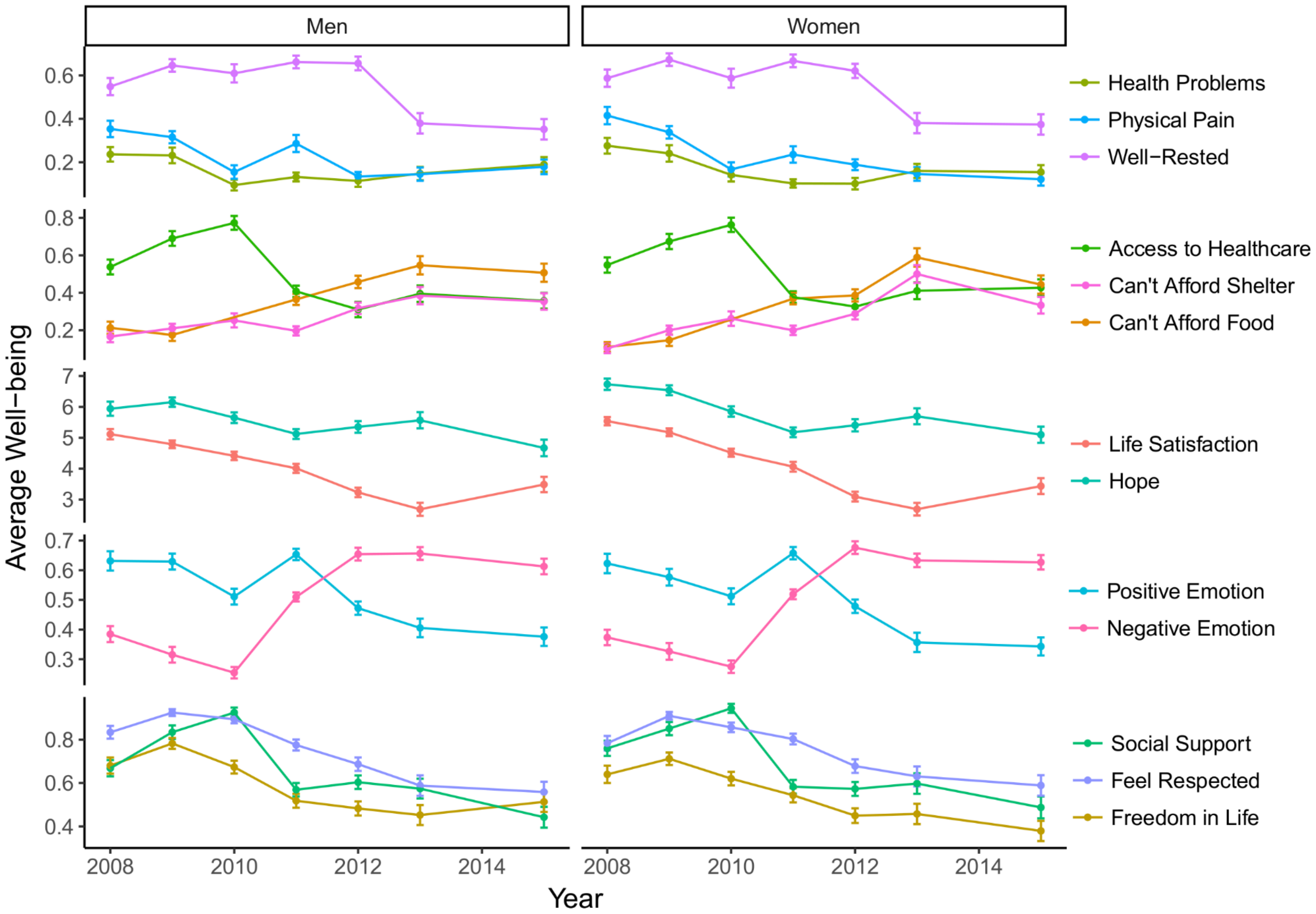


**Supplementary Figure 1**. **Sex-specific trends in well-being in Syria from 2008 to 2015.** Error bars indicate 95% CI (±1.96 * standard error). Source data are provided as a Source Data file.

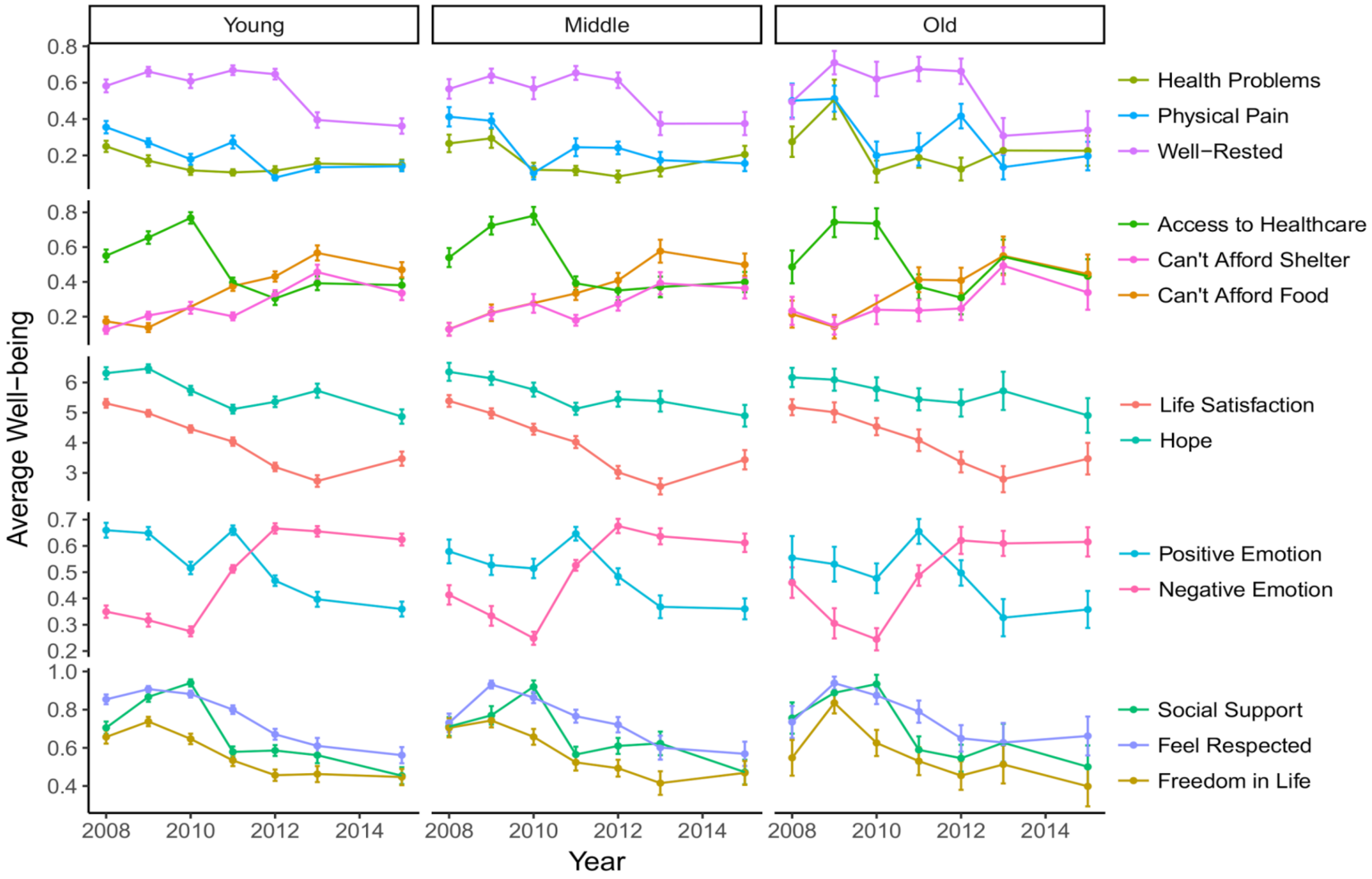


**Supplementary Figure 2. Age-specific trends in well-being in Syria from 2008 to 2015.** Younger adults, mid-life adults, and older adults refer to participants aged 35 or younger, participants aged 36-55, and participants 56 or older, respectively. Error bars indicate 95% CI (±1.96 * standard error). Source data are provided as a Source Data file.

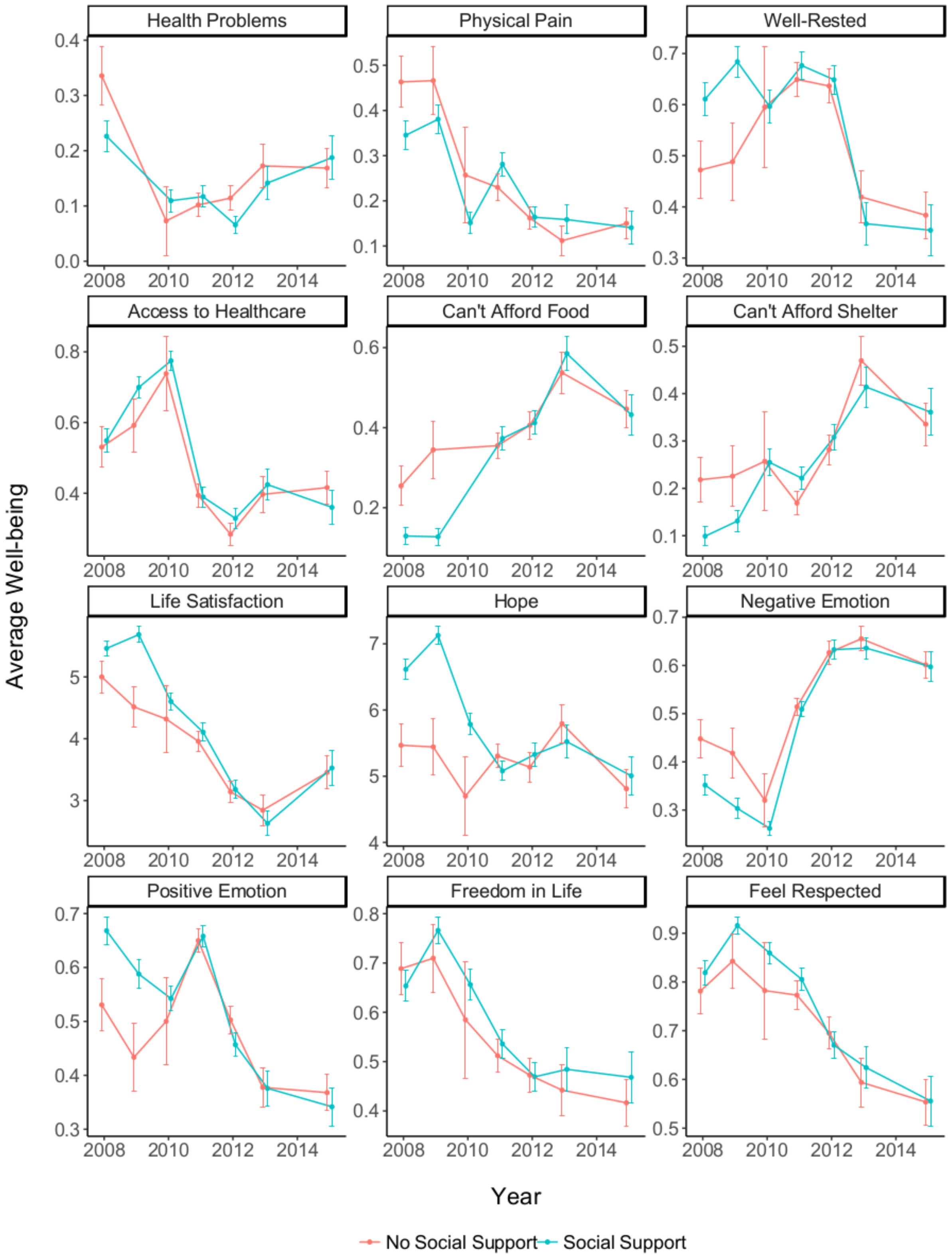


**Supplementary Figure 3. Temporal changes in well-being from 2008 to 2015 by availability of social support.** Error bars indicate 95% CI (±1.96 * standard error). Source data are provided as a Source Data file.

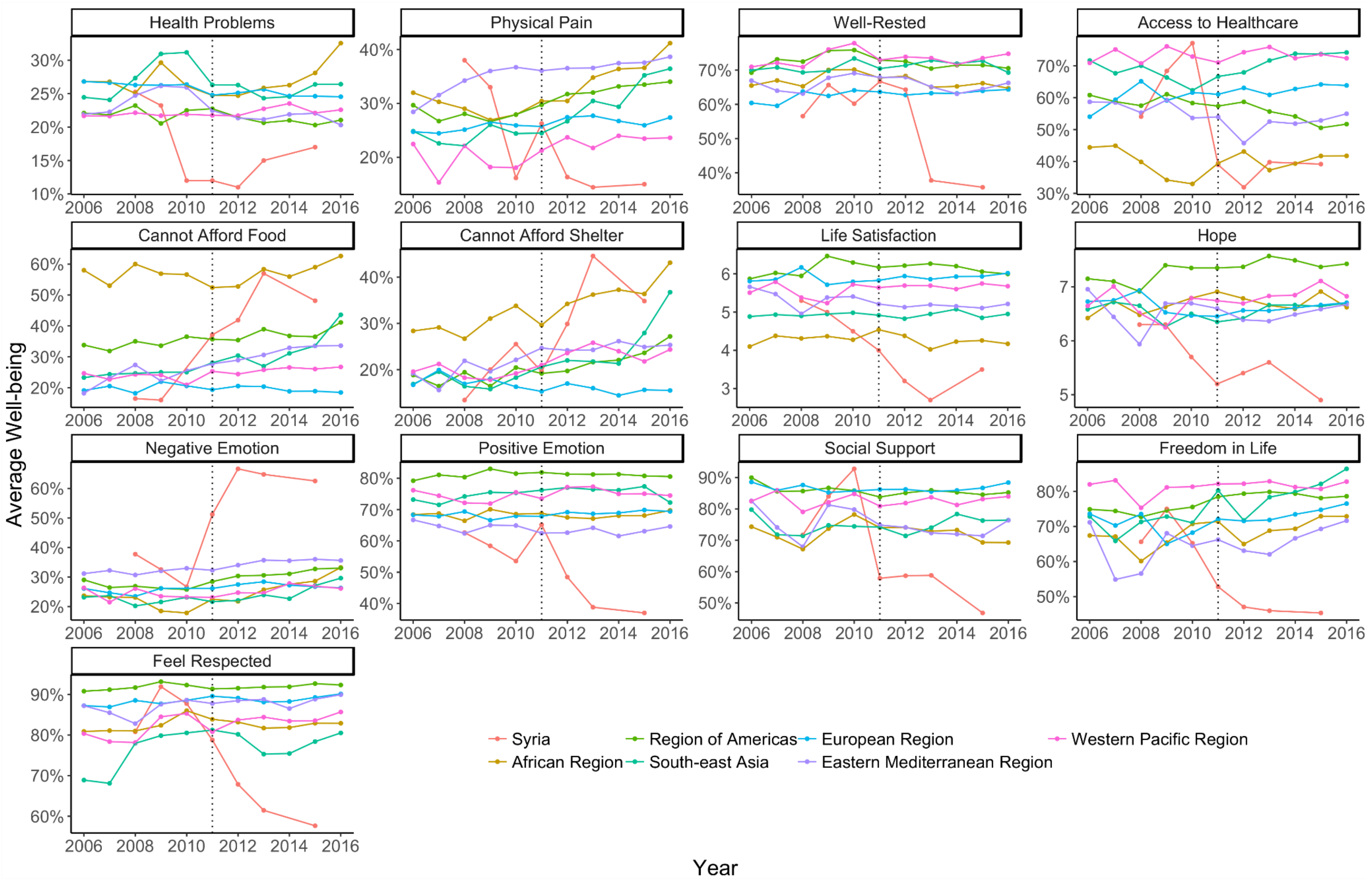


**Supplementary Figure 4. The longitudinal trends in physical, mental, and social well-being for Syria and 6 WHO regions from 2006 to 2016.** Source data are provided as a Source Data file.

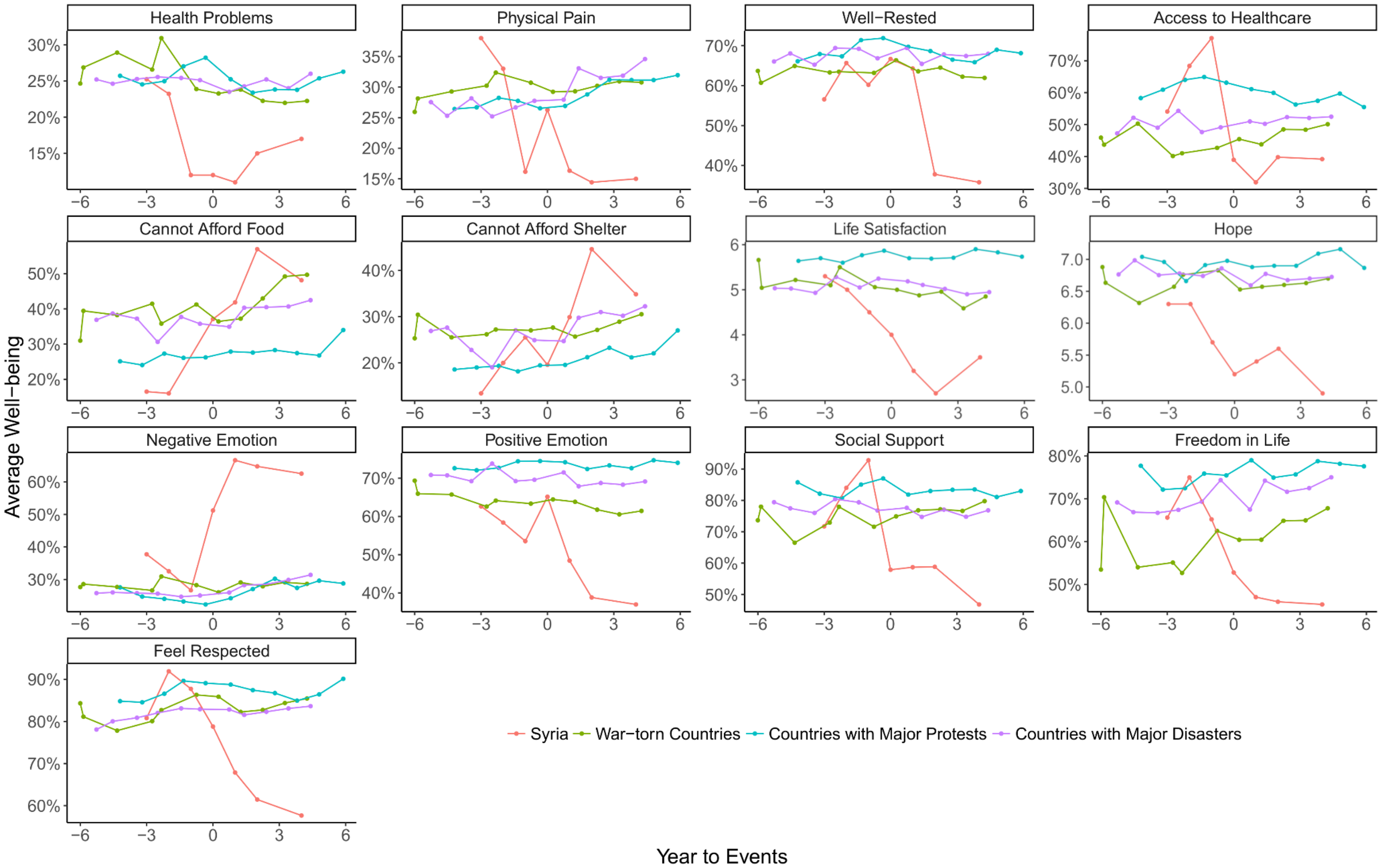


**Supplementary Figure 5. The longitudinal trends in well-being for Syria and countries that experienced war, protests, and disasters.** Source data are provided as a Source Data file.

**Supplementary Table 1.** Estimates and 95% confidence intervals from multilevel modeling of temporal changes in well-being in Syria.

| | Problems | Pain | Rested | Health Care | Cannot Afford Food | Cannot Afford Shelter | Life Sat. | Hope | Pos. Emot. | Neg. Emot. | Support | Respect | Freedom |
|---|---|---|---|---|---|---|---|---|---|---|---|---|---|
| **Overall Trends in Well-being** | | | | | | | | | | | | | |
| N | 8330 | 9319 | 9684 | 8039 | 7650 | 9783 | 11300 | 10263 | 8991 | 11393 | 8751 | 10726 | 10368 |
| Intercept | -1.354 | -0.615 | 0.773 | 0.520 | -1.527 | -1.670 | 5.152 | 6.222 | 0.648 | 0.299 | 1.380 | 0.914 | 2.186 |
| | [-1.508; -1.200] | [-0.734; -0.495] | [0.669; 0.877] | [0.381; 0.659] | [-1.658; -1.396] | [-1.807; -1.533] | [5.055; 5.248] | [6.111; 6.333] | [0.632; 0.664] | [0.281; 0.318] | [1.278; 1.483] | [0.824; 1.003] | [2.044; 2.327] |
| Year | -0.658 | -1.475 | -0.978 | -1.283 | 1.908 | 1.238 | -2.631 | -1.456 | -0.277 | 0.414 | -1.625 | -1.460 | -2.098 |
| | [-0.859; -0.456] | [-1.651; -1.300] | [-1.124; -0.831] | [-1.436; -1.131] | [1.735; 2.081] | [1.077; 1.398] | [-2.789; -2.473] | [-1.646; -1.267] | [-0.304; -0.251] | [0.393; 0.435] | [-1.790; -1.461] | [-1.607; -1.313] | [-2.269; -1.928] |
| **Estimates of Well-being based on the results from multilevel models** | | | | | | | | | | | | | |
| 2008 | 20.5% | 35.1% | 68.4% | 62.7% | 17.8% | 15.8% | 5.15 | 6.22 | 64.8% | 29.9% | 79.9% | 71.4% | 89.9% |
| 95% CI lower bound | 18.1% | 32.4% | 66.1% | 59.4% | 16.0% | 14.1% | 5.06 | 6.11 | 63.2% | 28.1% | 78.2% | 69.5% | 88.5% |
| 95% CI upper bound | 23.1% | 37.9% | 70.6% | 65.9% | 19.8% | 17.8% | 5.25 | 6.33 | 66.4% | 31.8% | 81.5% | 73.2% | 91.1% |
| 2015 | 11.8% | 11.0% | 44.9% | 31.8% | 59.4% | 39.4% | 2.52 | 4.77 | 37.1% | 71.3% | 43.9% | 36.7% | 52.2% |
| 95% CI lower bound | 9.9% | 9.4% | 41.3% | 28.6% | 55.2% | 35.6% | 2.36 | 4.58 | 34.4% | 69.2% | 39.9% | 33.3% | 47.9% |
| 95% CI upper bound | 14.1% | 12.8% | 48.6% | 35.2% | 63.5% | 43.2% | 2.68 | 4.96 | 39.7% | 73.4% | 48.0% | 40.2% | 56.4% |

**Supplementary Table 2.** Estimates and 95% confidence intervals from multilevel modeling of the demographic differences in temporal changes in well-being in Syria.

| | **Problems** | **Pain** | **Rested** | **Health Care** | **Cannot Afford Food** | **Cannot Afford Shelter** | **Life Sat.** | **Hope** | **Pos. Emot.** | **Neg. Emot.** | **Support** | **Respect** | **Freedom** |
|---|---|---|---|---|---|---|---|---|---|---|---|---|---|
| **Demographic Differences in Trends in Well-being** | | | | | | | | | | | | | |
| N | 8326 | 9314 | 9679 | 8036 | 7648 | 9778 | 11295 | 10258 | 8988 | 11388 | 8748 | 10721 | 10363 |
| Intercept | -1.434 | -0.707 | 0.742 | 0.566 | -1.362 | -1.590 | 4.983 | 6.033 | 0.654 | 0.295 | 1.273 | 0.989 | 2.324 |
| | [-1.618; -1.250] | [-0.855; -0.558] | [0.615; 0.868] | [0.405; 0.726] | [-1.522; -1.202] | [-1.749; -1.431] | [4.860; 5.105] | [5.890; 6.176] | [0.633; 0.674] | [0.273; 0.316] | [1.138; 1.408] | [0.875; 1.103] | [2.153; 2.495] |
| Year | -0.450 | -1.335 | -0.913 | -1.424 | 1.756 | 1.093 | -2.362 | -1.292 | -0.275 | 0.415 | -1.481 | -1.440 | -2.351 |
| | [-0.730; -0.171] | [-1.582; -1.089] | [-1.119; -0.706] | [-1.639; -1.209] | [1.518; 1.994] | [0.870; 1.316] | [-2.583; -2.141] | [-1.555; -1.029] | [-0.312; -0.238] | [0.385; 0.445] | [-1.708; -1.254] | [-1.645; -1.234] | [-2.594; -2.107] |
| Sex | 0.175 | 0.216 | 0.073 | -0.098 | -0.345 | -0.158 | 0.343 | 0.384 | -0.017 | 0.010 | 0.236 | -0.146 | -0.286 |
| | [-0.023; 0.374] | [0.060; 0.373] | [-0.073; 0.218] | [-0.256; 0.059] | [-0.543; -0.146] | [-0.331; 0.015] | [0.189; 0.498] | [0.195; 0.573] | [-0.043; 0.009] | [-0.011; 0.032] | [0.056; 0.417] | [-0.291; -0.001] | [-0.472; -0.100] |
| Age | 0.115 | 0.146 | 0.043 | -0.033 | 0.096 | 0.036 | 0.014 | -0.004 | -0.023 | 0.011 | 0.072 | 0.016 | -0.054 |
| | [0.022; 0.208] | [0.072; 0.220] | [-0.027; 0.113] | [-0.110; 0.043] | [0.003; 0.189] | [-0.044; 0.117] | [-0.060; 0.089] | [-0.094; 0.086] | [-0.036; -0.011] | [0.001; 0.022] | [-0.014; 0.157] | [-0.053; 0.086] | [-0.144; 0.036] |
| Year X Sex | -0.460 | -0.399 | -0.144 | 0.297 | 0.327 | 0.282 | -0.554 | -0.328 | 0.003 | -0.006 | -0.320 | -0.054 | 0.532 |
| | [-0.865; -0.055] | [-0.753; -0.044] | [-0.437; 0.149] | [-0.008; 0.601] | [-0.022; 0.676] | [-0.040; 0.604] | [-0.869; -0.239] | [-0.706; 0.049] | [-0.049; 0.056] | [-0.048; 0.037] | [-0.651; 0.012] | [-0.349; 0.241] | [0.190; 0.873] |
| Year X Age | -0.039 | 0.113 | -0.143 | 0.085 | -0.188 | -0.010 | -0.048 | -0.036 | 0.035 | -0.019 | -0.024 | -0.028 | 0.131 |
| | [-0.219; 0.141] | [-0.040; 0.265] | [-0.282; -0.005] | [-0.058; 0.228] | [-0.350; -0.026] | [-0.157; 0.138] | [-0.196; 0.100] | [-0.212; 0.140] | [0.010; 0.059] | [-0.039; 0.001] | [-0.177; 0.129] | [-0.165; 0.109] | [-0.032; 0.293] |
| Sex X Age | -0.031 | 0.013 | -0.080 | 0.083 | -0.149 | -0.024 | -0.031 | -0.108 | -0.004 | -0.000 | -0.075 | 0.035 | -0.051 |
| | [-0.164; 0.103] | [-0.093; 0.119] | [-0.181; 0.020] | [-0.025; 0.192] | [-0.287; -0.010] | [-0.142; 0.095] | [-0.137; 0.076] | [-0.237; 0.022] | [-0.022; 0.014] | [-0.015; 0.015] | [-0.199; 0.049] | [-0.066; 0.135] | [-0.176; 0.074] |
| Year X Sex X Age | 0.100 | -0.104 | 0.228 | -0.103 | 0.274 | -0.092 | 0.011 | 0.259 | 0.006 | -0.005 | -0.013 | -0.039 | 0.072 |
| | [-0.162; 0.361] | [-0.327; 0.119] | [0.031; 0.425] | [-0.307; 0.101] | [0.034; 0.513] | [-0.309; 0.124] | [-0.201; 0.223] | [0.006; 0.512] | [-0.029; 0.041] | [-0.034; 0.024] | [-0.236; 0.210] | [-0.237; 0.159] | [-0.155; 0.300] |

**Supplementary Table 3.** Estimated changes in physical well-being in 13 Syrian governorates based on the random effects from multilevel models.

| | Health Problems | | Pain | | Rested | | Health Care | | Cannot Afford Food | | Cannot Afford Shelter | |
|---|---|---|---|---|---|---|---|---|---|---|---|---|
| Likelihood Ratio Test (χ2, df = 2) | 32.1 | | 24.5 | | 33.7 | | 74.1 | | 10.9 | | 35.6 | |
| p-value | <0.0001 | | <0.0001 | | <0.0001 | | <0.0001 | | 0.0043 | | <0.0001 | |
| | **OR** | **95% CI** | **OR** | **95% CI** | **OR** | **95% CI** | **OR** | **95% CI** | **OR** | **95% CI** | **OR** | **95% CI** |
| Damascus | 0.379 | [0.339,0.423] | 0.218 | [0.192,0.246] | 0.197 | [0.178,0.219] | 0.666 | [0.593,0.748] | 4.188 | [4.0,4.39] | 1.778 | [1.63,1.94] |
| Rural Damascus | 0.508 | [0.453,0.570] | 0.265 | [0.241,0.291] | 0.204 | [0.188,0.221] | 0.064 | [0.057,0.072] | 7.595 | [7.35,7.85] | 3.662 | [3.39,3.96] |
| Sweida | 0.053 | [0.038,0.073] | 0.029 | [0.020,0.043] | 0.420 | [0.317,0.556] | 0.491 | [0.329,0.735] | 7.711 | [6.77,8.78] | 10.269 | [7.01,15.1] |
| Daraa | 0.324 | [0.251,0.418] | 0.400 | [0.318,0.503] | 0.316 | [0.266,0.375] | 0.120 | [0.093,0.154] | 6.886 | [6.30,7.52] | 2.374 | [2.04,2.76] |
| Homs | 0.310 | [0.283,0.338] | 0.243 | [0.218,0.271] | 0.530 | [0.484,0.580] | 0.204 | [0.178,0.234] | 7.439 | [7.11,7.78] | 5.382 | [4.86,5.96] |
| Hama | 1.125 | [0.934,1.36] | 0.113 | [0.099,0.129] | 0.718 | [0.645,0.799] | 0.152 | [0.131,0.177] | 14.905 | [14.,15.9] | 9.045 | [7.87,10.4] |
| Tartus | 0.453 | [0.376,0.545] | 0.119 | [0.100,0.140] | 0.975 | [0.847,1.12] | 1.327 | [1.08,1.64] | 10.338 | [9.48,11.3] | 6.924 | [5.67,8.46] |
| Latakia | 0.295 | [0.251,0.348] | 0.353 | [0.296,0.421] | 0.311 | [0.269,0.359] | 0.758 | [0.630,0.910] | 5.839 | [5.46,6.25] | 8.536 | [7.11,10.3] |
| Aleppo | 0.464 | [0.445,0.483] | 0.220 | [0.210,0.231] | 0.531 | [0.512,0.552] | 0.317 | [0.303,0.331] | 5.824 | [5.73,5.92] | 3.611 | [3.47,3.76] |
| Idlib | 1.439 | [1.18,1.76] | 0.246 | [0.214,0.284] | 0.286 | [0.256,0.321] | 0.209 | [0.177,0.247] | 4.995 | [4.75,5.25] | 2.566 | [2.30,2.86] |
| Deir ez-zor | 0.732 | [0.608,0.880] | 0.190 | [0.163,0.222] | 0.413 | [0.364,0.469] | 0.319 | [0.267,0.380] | 6.979 | [6.56,7.42] | 2.078 | [1.86,2.33] |
| Al- Hasakah | 0.978 | [0.764,1.25] | 0.490 | [0.403,0.595] | 0.270 | [0.234,0.312] | 0.202 | [0.165,0.246] | 4.755 | [4.48,5.05] | 1.335 | [1.19,1.50] |
| Ar-Raqqah | 1.768 | [1.22,2.56] | 0.541 | [0.414,0.706] | 0.128 | [0.105,0.157] | 0.693 | [0.548,0.876] | 7.855 | [7.16,8.61] | 4.120 | [3.40,4.99] |

**Supplementary Table 4.** Estimated changes in mental and social well-being in 13 Syrian governorates based on the random effects from multilevel models.

| | Life Satisfaction | | Hope | | Pos. Emot. | | Neg. Emot. | | Support | | Respect | | Freedom | |
|---|---|---|---|---|---|---|---|---|---|---|---|---|---|---|
| Likelihood Ratio Test (χ2, df = 2) | 9.2 | | 38.1 | | 8.6 | | 36.2 | | 14.6 | | 42.1 | | 12.7 | |
| p-value | 0.0102 | | <0.0001 | | 0.0135 | | <0.0001 | | 0.0007 | | <0.0001 | | 0.002 | |
| | **b** | **95% CI** | **b** | **95% CI** | **b** | **95% CI** | **b** | **95% CI** | **OR** | **95% CI** | **OR** | **95% CI** | **OR** | **95% CI** |
| Damascus | -2.442 | [-2.75,-2.69] | -1.497 | [-1.66,-1.34] | -0.257 | [-0.258,-0.255] | 0.446 | [0.445,0.448] | 0.125 | [0.110,0.142] | 0.129 | [0.114,0.146] | 0.334 | [0.317,0.351] |
| Rural Damascus | -2.085 | [-2.40,-2.35] | -1.317 | [-1.45,-1.18] | -0.307 | [-0.308,-0.306] | 0.458 | [0.456,0.459] | 0.110 | [0.100,0.122] | 0.056 | [0.050,0.062] | 0.295 | [0.284,0.308] |
| Sweida | -2.372 | [-2.77,-2.59] | -1.388 | [-1.97,-0.809] | -0.281 | [-0.285,-0.277] | 0.346 | [0.342,0.351] | 0.381 | [0.286,0.507] | 0.083 | [0.055,0.125] | 0.206 | [0.180,0.235] |
| Daraa | -2.479 | [-2.89,-2.78] | -0.550 | [-0.841,-0.259] | -0.275 | [-0.278,-0.272] | 0.472 | [0.469,0.475] | 0.190 | [0.155,0.233] | 0.126 | [0.102,0.156] | 0.222 | [0.204,0.243] |
| Homs | -1.904 | [-2.21,-2.10] | -1.572 | [-1.76,-1.38] | -0.293 | [-0.295,-0.292] | 0.347 | [0.345,0.348] | 0.261 | [0.233,0.292] | 0.090 | [0.079,0.102] | 0.300 | [0.286,0.314] |
| Hama | -2.127 | [-2.48,-2.41] | 0.042 | [-0.149,0.234] | -0.216 | [-0.218,-0.214] | 0.408 | [0.406,0.410] | 0.162 | [0.141,0.186] | 0.077 | [0.066,0.091] | 0.148 | [0.139,0.158] |
| Tartus | -2.075 | [-2.47,-2.34] | -1.314 | [-1.60,-1.03] | -0.178 | [-0.180,-0.175] | 0.363 | [0.361,0.366] | 0.238 | [0.200,0.283] | 0.791 | [0.692,0.904] | 0.315 | [0.291,0.340] |
| Latakia | -2.324 | [-2.66,-2.56] | -1.084 | [-1.34,-0.829] | -0.356 | [-0.359,-0.354] | 0.418 | [0.415,0.420] | 0.324 | [0.274,0.382] | 0.215 | [0.182,0.254] | 0.146 | [0.134,0.158] |
| Aleppo | -2.614 | [-2.79,-2.76] | -2.222 | [-2.29,-2.16] | -0.259 | [-0.260,-0.258] | 0.333 | [0.332,0.334] | 0.166 | [0.158,0.175] | 0.163 | [0.156,0.171] | 0.177 | [0.173,0.181] |
| Idlib | -2.438 | [-2.72,-2.63] | -1.775 | [-1.99,-1.56] | -0.298 | [-0.299,-0.296] | 0.489 | [0.487,0.491] | 0.259 | [0.225,0.298] | 0.148 | [0.130,0.170] | 0.166 | [0.156,0.178] |
| Deir ez-zor | -2.650 | [-3.06,-2.96] | -2.075 | [-2.35,-1.80] | -0.281 | [-0.283,-0.279] | 0.448 | [0.446,0.451] | 0.600 | [0.517,0.697] | 0.056 | [0.046,0.067] | 0.258 | [0.241,0.276] |
| Al- Hasakah | -2.284 | [-2.64,-2.56] | -1.192 | [-1.46,-0.923] | -0.367 | [-0.369,-0.364] | 0.521 | [0.519,0.523] | 0.261 | [0.221,0.307] | 0.069 | [0.057,0.082] | 0.299 | [0.279,0.321] |
| Ar-Raqqah | -2.505 | [-2.94,-2.83] | -0.195 | [-0.552,0.163] | -0.297 | [-0.300,-0.294] | 0.568 | [0.564,0.571] | 0.128 | [0.102,0.162] | 0.139 | [0.111,0.175] | 0.307 | [0.279,0.337] |

**Supplementary Table 5.** Odd ratios and 95% confidence intervals comparing the levels of well-being of participants who were exposed and not exposed to the Syria conflict.

| | **Problems** | **Pain** | **Rested** | **Health Care** | **Cannot Afford Food** | **Cannot Afford Shelter** | **Life Sat.** | **Hope** | **Pos. Emot.** | **Neg. Emot.** | **Support** | **Respect** | **Freedom** |
|---|---|---|---|---|---|---|---|---|---|---|---|---|---|
| **<u>Exposed vs Not Exposed (Reference Group) to the Syria Conflict</u>** | | | | | | | | | | | | | |
| OR/b | 1.038 | 0.986 | 1.030 | 1.074 | 1.362 | 1.235 | -0.170 | -0.327 | 0.002 | 0.013 | 0.999 | 0.820 | 1.078 |
| 95% CI | [0.817, 1.319] | [0.766, 1.271] | [0.835, 1.270] | [0.885, 1.303] | [1.108, 1.674] | [1.012, 1.508] | [-0.404, 0.064] | [-0.597, -0.057] | [-0.029, 0.034] | [-0.011, 0.037] | [0.815, 1.225] | [0.672, 1.002] | [0.889, 1.307] |

**Supplementary Table 6.** Estimates and 95% confidence intervals from multiple imputations of multilevel modeling testing the differences in well-being by exposure to the Syria conflict.

| | **Problems** | **Pain** | **Rested** | **Health Care** | **Cannot Afford Food** | **Cannot Afford Shelter** | **Life Sat.** | **Hope** | **Pos. Emot.** | **Neg. Emot.** | **Support** | **Respect** | **Freedom** |
|---|---|---|---|---|---|---|---|---|---|---|---|---|---|
| Intercept | -1.775 [-2.038, -1.511] | -1.777 [-2.101, -1.453] | -0.501 [-0.742, -0.260] | -0.475 [-0.819, -0.132] | 0.050 [-0.170, 0.270] | -0.518 [-0.749, -0.288] | 2.751 [2.497, 3.005] | 5.709 [5.365, 6.053] | 0.404 [0.371, 0.438] | 0.653 [0.627, 0.678] | 0.292 [0.053, 0.532] | 0.459 [0.241, 0.677] | -0.211 [-0.427, 0.006] |
| Year | 0.245 [-0.092, 0.583] | 0.173 [-0.177, 0.524] | -0.088 [-0.386, 0.210] | -0.174 [-0.449, 0.100] | -0.199 [-0.475, 0.076] | -0.248 [-0.529, 0.034] | 0.837 [0.510, 1.165] | -0.818 [-1.201, -0.436] | -0.030 [-0.074, 0.015] | -0.056 [-0.089, -0.023] | -0.569 [-0.843, -0.294] | -0.105 [-0.384, 0.173] | 0.193 [-0.073, 0.459] |
| Sex | 0.057 [-0.289, 0.404] | -0.016 [-0.374, 0.342] | 0.014 [-0.270, 0.297] | 0.086 [-0.184, 0.357] | 0.114 [-0.166, 0.394] | 0.418 [0.140, 0.695] | 0.023 [-0.302, 0.347] | 0.164 [-0.214, 0.542] | -0.047 [-0.091, -0.003] | -0.025 [-0.058, 0.008] | 0.075 [-0.199, 0.350] | 0.163 [-0.113, 0.440] | -0.026 [-0.298, 0.246] |
| Age | -0.083 [-0.242, 0.077] | 0.058 [-0.089, 0.206] | -0.055 [-0.182, 0.073] | 0.010 [-0.103, 0.123] | -0.041 [-0.159, 0.077] | 0.055 [-0.062, 0.173] | -0.072 [-0.210, 0.067] | 0.010 [-0.157, 0.177] | -0.010 [-0.030, 0.009] | 0.000 [-0.014, 0.015] | 0.060 [-0.058, 0.177] | 0.017 [-0.102, 0.135] | -0.003 [-0.116, 0.110] |
| Exposure | 0.037 [-0.203, 0.277] | -0.014 [-0.267, 0.240] | 0.029 [-0.180, 0.239] | 0.071 [-0.122, 0.264] | 0.309 [0.103, 0.515] | 0.211 [0.012, 0.411] | -0.170 [-0.404, 0.064] | -0.327 [-0.597,- 0.057] | 0.002 [-0.029, 0.034] | 0.013 [-0.011, 0.037] | -0.001 [-0.205, 0.203] | -0.198 [-0.398, 0.002] | 0.075 [-0.118, 0.268] |
| Year X Sex | -0.243 [-0.727, 0.240] | -0.410 [-0.920, 0.100] | 0.061 [-0.343, 0.466] | 0.284 [-0.099, 0.666] | -0.317 [-0.704, 0.070] | -0.504 [-0.896, -0.111] | -0.092 [-0.555, 0.371] | 0.242 [-0.294, 0.779] | 0.015 [-0.047, 0.078] | 0.037 [-0.010, 0.085] | 0.086 [-0.300, 0.473] | -0.040 [-0.429, 0.349] | -0.489 [-0.872, -0.106] |
| Year X Age | 0.303 [0.093, 0.513] | 0.121 [-0.084, 0.325] | 0.030 [-0.155, 0.216] | 0.030 [-0.137, 0.196] | -0.035 [-0.214, 0.145] | 0.001 [-0.168, 0.170] | 0.071 [-0.135, 0.277] | -0.125 [-0.361, 0.110] | 0.011 [-0.017, 0.039] | 0.003 [-0.018, 0.024] | 0.051 [-0.120, 0.222] | 0.068 [-0.107, 0.243] | -0.061 [-0.225, 0.103] |
| Sex X Age | 0.271 [0.059, 0.482] | -0.056 [-0.283, 0.172] | 0.013 [-0.171, 0.198] | 0.132 [-0.033, 0.297] | 0.009 [-0.166, 0.184] | -0.107 [-0.272, 0.059] | 0.059 [-0.142, 0.260] | -0.050 [-0.286, 0.186] | -0.014 [-0.042, 0.013] | -0.008 [-0.029, 0.012] | 0.012 [-0.159, 0.184] | -0.022 [-0.196, 0.152] | 0.039 [-0.125, 0.203] |
| Year X Sex X Age | -0.384 [-0.680, -0.088] | -0.175 [-0.504, 0.153] | 0.045 [-0.214, 0.305] | -0.119 [-0.359, 0.121] | 0.122 [-0.136, 0.380] | -0.025 [-0.271, 0.220] | -0.137 [-0.434, 0.161] | 0.285 [-0.058, 0.627] | 0.023 [-0.017, 0.063] | -0.001 [-0.031, 0.029] | -0.168 [-0.424, 0.089] | 0.033 [-0.223, 0.289] | 0.038 [-0.205, 0.281] |

**Supplementary Table 7.** Estimates and 95% confidence intervals from multilevel modeling testing the differences in well-being by exposure to the Syria conflict.

| | Problems | Pain | Rested | Health Care | Cannot Afford Food | Cannot Afford Shelter | Life Sat. | Hope | Pos. Emot. | Neg. Emot. | Support | Respect | Freedom |
|---|---|---|---|---|---|---|---|---|---|---|---|---|---|
| **Differences in Well-being by Exposure Status (2013-2015)** | | | | | | | | | | | | | |
| N | 2024 | 1995 | 1657 | 1913 | 1626 | 1742 | 1951 | 1846 | 2024 | 2024 | 1673 | 1747 | 1707 |
| Intercept | -1.775 | -1.773 | -0.508 | -0.474 | 0.058 | -0.579 | 2.751 | 5.725 | 0.404 | 0.653 | 0.299 | 0.448 | -0.216 |
| | [-2.038; -1.511] | [-2.096; -1.451] | [-0.742; -0.275] | [-0.817; -0.130] | [-0.152; 0.268] | [-0.809; -0.349] | [2.497; 3.004] | [5.385; 6.066] | [0.371; 0.438] | [0.627; 0.678] | [0.073; 0.525] | [0.237; 0.659] | [-0.423; -0.008] |
| Year | 0.245 | 0.169 | -0.109 | -0.185 | -0.196 | -0.174 | 0.839 | -0.828 | -0.030 | -0.056 | -0.573 | -0.099 | 0.239 |
| | [-0.092; 0.583] | [-0.182; 0.520] | [-0.399; 0.181] | [-0.458; 0.087] | [-0.474; 0.081] | [-0.453; 0.105] | [0.513; 1.165] | [-1.201; -0.456] | [-0.074; 0.015] | [-0.089; -0.023] | [-0.849; -0.296] | [-0.372; 0.174] | [-0.028; 0.505] |
| Sex | 0.057 | -0.007 | 0.010 | 0.069 | 0.168 | 0.475 | 0.020 | 0.159 | -0.047 | -0.025 | 0.071 | 0.178 | 0.014 |
| | [-0.289; 0.404] | [-0.364; 0.350] | [-0.271; 0.292] | [-0.200; 0.339] | [-0.113; 0.448] | [0.200; 0.750] | [-0.303; 0.343] | [-0.211; 0.530] | [-0.091; -0.003] | [-0.058; 0.008] | [-0.202; 0.344] | [-0.098; 0.454] | [-0.252; 0.279] |
| Age | -0.083 | 0.059 | -0.067 | 0.007 | -0.038 | 0.070 | -0.073 | 0.015 | -0.010 | 0.000 | 0.061 | 0.004 | 0.003 |
| | [-0.242; 0.077] | [-0.089; 0.208] | [-0.193; 0.058] | [-0.107; 0.122] | [-0.159; 0.083] | [-0.050; 0.190] | [-0.212; 0.065] | [-0.149; 0.178] | [-0.030; 0.009] | [-0.014; 0.015] | [-0.056; 0.179] | [-0.116; 0.123] | [-0.110; 0.115] |
| Exposure | 0.037 | -0.010 | 0.030 | 0.080 | 0.327 | 0.202 | -0.176 | -0.333 | 0.002 | 0.013 | -0.011 | -0.212 | 0.059 |
| | [-0.203; 0.277] | [-0.263; 0.243] | [-0.173; 0.234] | [-0.111; 0.272] | [0.127; 0.526] | [0.005; 0.399] | [-0.408; 0.057] | [-0.597; -0.069] | [-0.029; 0.034] | [-0.011; 0.037] | [-0.208; 0.186] | [-0.408; -0.017] | [-0.133; 0.251] |
| Year X Sex | -0.243 | -0.418 | 0.090 | 0.306 | -0.409 | -0.568 | -0.094 | 0.250 | 0.015 | 0.037 | 0.117 | -0.045 | -0.562 |
| | [-0.727; 0.240] | [-0.928; 0.091] | [-0.314; 0.493] | [-0.074; 0.687] | [-0.804; -0.013] | [-0.960; -0.176] | [-0.555; 0.367] | [-0.274; 0.774] | [-0.047; 0.078] | [-0.010; 0.085] | [-0.276; 0.510] | [-0.434; 0.344] | [-0.943; -0.181] |
| Year X Age | 0.303 | 0.120 | 0.047 | 0.034 | -0.066 | -0.004 | 0.073 | -0.141 | 0.011 | 0.003 | 0.062 | 0.078 | -0.071 |
| | [0.093; 0.513] | [-0.085; 0.325] | [-0.135; 0.230] | [-0.134; 0.201] | [-0.242; 0.110] | [-0.176; 0.168] | [-0.131; 0.277] | [-0.374; 0.092] | [-0.017; 0.039] | [-0.018; 0.024] | [-0.109; 0.233] | [-0.096; 0.252] | [-0.236; 0.095] |
| Sex X Age | 0.271 | -0.059 | 0.022 | 0.145 | 0.003 | -0.127 | 0.066 | -0.059 | -0.014 | -0.008 | 0.016 | -0.031 | 0.033 |
| | [0.059; 0.482] | [-0.281; 0.164] | [-0.158; 0.201] | [-0.021; 0.311] | [-0.173; 0.180] | [-0.296; 0.042] | [-0.134; 0.267] | [-0.291; 0.174] | [-0.042; 0.013] | [-0.029; 0.012] | [-0.157; 0.189] | [-0.205; 0.143] | [-0.129; 0.195] |
| Year X Sex X Age | -0.384 | -0.173 | 0.045 | -0.133 | 0.174 | -0.023 | -0.153 | 0.302 | 0.023 | -0.001 | -0.216 | 0.049 | 0.046 |
| | [-0.680; -0.088] | [-0.498; 0.152] | [-0.214; 0.304] | [-0.374; 0.107] | [-0.082; 0.430] | [-0.274; 0.228] | [-0.448; 0.142] | [-0.032; 0.636] | [-0.017; 0.063] | [-0.031; 0.029] | [-0.468; 0.037] | [-0.203; 0.301] | [-0.196; 0.287] |

**Supplementary Table 8.** Summary of the additive and multiplicative interaction terms testing social support as an effect modifier of temporal changes in well-being.

| | Year | | | | | |
|---|---|---|---|---|---|---|
| | 2008 | | | 2015 | | |
| | N | OR | 95%CI | N | OR | 95%CI |
| Health Problems | | | | | | |
| No Social Support | 81/300 | Reference | | 71/429 | 0.63 | (0.42, 0.94) |
| Social Support | 186/885 | 0.68 | (0.52, 0.88) | 69/368 | 0.58 | (0.36, 0.95) |
| Measure of interaction on additive scale | | | | RERI (95% CI) = | | 0.28 (-0.04, 0.59) |
| Measure of interaction on multiplicative scale | | | | Ratio of ORs (95% CI) = | | 1.37 (0.88, 1.86) |
| Physical Pain | | | | | | |
| No Social Support | 120/299 | Reference | | 61/429 | 0.17 | (0.12, 0.24) |
| Social Support | 289/884 | 0.69 | (0.56, 0.84) | 51/368 | 0.19 | (0.12, 0.28) |
| Measure of interaction on additive scale | | | | RERI (95% CI) = | | 0.33 (0.16,0.49) |
| Measure of interaction on multiplicative scale | | | | Ratio of ORs (95% CI) = | | 1.59 (1.17,2.01) |
| Well-restedness | | | | | | |
| No Social Support | 177/298 | Reference | | 141/358 | 0.65 | (0.48, 0.88) |
| Social Support | 568/874 | 1.46 | (1.22, 1.75) | 109/295 | 0.57 | (0.40, 0.80) |
| Measure of interaction on additive scale | | | | RERI (95% CI) = | | -0.54 (-0.90, -0.18) |
| Measure of interaction on multiplicative scale | | | | Ratio of ORs (95% CI) = | | 0.60 (0.26, 0.94) |
| Health Care | | | | | | |
| No Social Support | 158/297 | Reference | | 171/418 | 0.43 | (0.32, 0.58) |
| Social Support | 520/869 | 1.82 | (1.52, 2.19) | 130/353 | 0.40 | (0.28, 0.56) |
| Measure of interaction on additive scale | | | | RERI (95% CI) = | | -0.86 (-1.24, -0.48) |
| Measure of interaction on multiplicative scale | | | | Ratio of ORs (95% CI) = | | 0.50 (0.15, 0.85) |
| Food | | | | | | |
| No Social Support | 60/294 | Reference | | 162/346 | 2.80 | (2.03, 3.87) |
| Social Support | 90/881 | 0.49 | (0.40, 0.61) | 135/298 | 3.95 | (2.70, 5.77) |
| Measure of interaction on additive scale | | | | RERI (95% CI) = | | 1.65 (0.59, 2.72) |
| Measure of interaction on multiplicative scale | | | | Ratio of ORs (95% CI) = | | 2.86 (2.48, 3.24) |
| Shelter | | | | | | |
| No Social Support | 54/295 | Reference | | 129/386 | 2.20 | (1.56, 3.11) |
| Social Support | 76/881 | 0.68 | (0.55, 0.86) | 121/325 | 3.28 | (2.23, 4.83) |
| Measure of interaction on additive scale | | | | RERI (95% CI) = | | 1.39 (0.73, 2.05) |
| Measure of interaction on multiplicative scale | | | | Ratio of ORs (95% CI) = | | 2.18 (1.79, 2.56) |
| Respect | | | | | | |
| No Social Support | 242/294 | Reference | | 204/366 | 0.20 | (0.14, 0.28) |
| Social Support | 744/871 | 1.65 | (1.32, 2.06) | 175/315 | 0.18 | (0.12, 0.27) |
| Measure of interaction on additive scale | | | | RERI (95% CI) = | | -0.67 (-1.06, -0.28) |
| Measure of interaction on multiplicative scale | | | | Ratio of ORs (95% CI) = | | 0.54 (0.15, 0.93) |

| | | | | | | |
|---|---|---|---|---|---|---|
| <u>Freedom</u> | | | | | | |
| No Social Support | 180/278 | Reference | | 159/374 | 0.32 | (0.23, 0.44) |
| Social Support | 517/854 | 1.24 | (1.03, 1.50) | 136/309 | 0.34 | (0.24, 0.48) |
| Measure of interaction on additive scale | | | | RERI (95% CI) = | | -0.22 (-0.50, 0.05) |
| Measure of interaction on multiplicative scale | | | | Ratio of ORs (95% CI) = | | 0.85 (0.50, 1.20) |

**Supplementary Table 9.** Estimates and 95% confidence intervals from multiple imputations of multilevel models testing social support as an effect modifier of temporal changes in well-being in Syria.

| | **Problems** | **Pain** | **Rested** | **Health Care** | **Cannot Afford Food** | **Cannot Afford Shelter** | **Life Sat.** | **Hope** | **Pos. Emot.** | **Neg. Emot.** | **Respect** | **Freedom** |
|---|---|---|---|---|---|---|---|---|---|---|---|---|
| Intercept | -1.336 [-1.602, -1.069] | -0.350 [-0.555, -0.146] | 0.416 [0.233, 0.598] | 0.152 [-.062, 0.365] | -0.943 [-1.153, -0.734] | -1.433 [-1.655, -1.211] | 4.474 [4.272, 4.677] | 5.425 [5.170, 5.681] | 0.610 [0.579, 0.641] | 0.434 [0.406, 0.461] | 1.718 [1.482, 1.953] | 0.699 [0.515, 0.882] |
| Year | -0.469 [-0.873, -0.065] | -1.775 [-2.136, -1.415] | -0.433 [-0.741, -0.126] | -0.838 [-1.136, -0.539] | 1.031 [0.709, 1.352] | 0.790 [0.445, 1.134] | -1.598 [-1.938, -1.258] | -0.505 [-0.921, -0.089] | -0.191 [-0.243, -0.139] | 0.233 [0.190, 0.275] | -1.604 [-1.947, -1.261] | -1.145 [-1.458, -0.832] |
| Sex | 0.250 [0.015, 0.485] | 0.236 [0.062, 0.409] | 0.047 [-0.117, 0.212] | -0.126 [-0.286, 0.035] | -0.263 [-0.467, -0.058] | -0.306 [-0.512, -0.101] | 0.299 [0.117, 0.480] | 0.369 [0.145, 0.593] | 0.002 [-0.026, 0.031] | 0.013 [-0.010, 0.036] | -0.278 [-0.485, -0.071] | -0.060 [-0.229, 0.110] |
| Age | -0.037 [-0.151, 0.077] | 0.166 [0.083, 0.249] | 0.037 [-0.042, 0.116] | -0.043 [-0.120, 0.035] | 0.096 [0.003, 0.189] | 0.047 [-0.045, 0.140] | 0.013 [-0.075, 0.101] | 0.024 [-0.082, 0.129] | -0.012 [-0.025, 0.002] | 0.014 [0.003, 0.026] | -0.095 [-0.195, 0.004] | -0.008 [-0.089, 0.073] |
| Support | -0.386 [-0.645, -0.126] | -0.373 [-0.576, -0.170] | 0.379 [0.196, 0.561] | 0.601 [0.416, 0.786] | -0.709 [-0.926, -0.492] | -0.379 [-0.602, -0.156] | 0.893 [0.682, 1.104] | 0.980 [0.719, 1.242] | 0.057 [0.025, 0.090] | -0.158 [-0.185, -0.132] | 0.500 [0.275, 0.724] | 0.215 [0.025, 0.406] |
| Year X Sex | -0.550 [-0.989, -0.110] | -0.429 [-0.800, -0.058] | -0.072 [-0.398, 0.254] | 0.322 [0.011, 0.633] | 0.247 [-0.107, 0.600] | 0.451 [0.079, 0.823] | -0.488 [-0.830, -0.146] | -0.289 [-0.701, 0.123] | -0.023 [-0.076, 0.029] | -0.011 [-0.054, 0.032] | 0.505 [0.140, 0.869] | -0.171 [-0.502, 0.160] |
| Year X Age | 0.173 [-0.026, 0.372] | 0.081 [-0.081, 0.243] | -0.163 [-0.317, -0.009] | 0.093 [-0.051, 0.237] | -0.176 [-0.337, -0.016] | -0.034 [-0.200, 0.132] | -0.040 [-0.203, 0.123] | -0.075 [-0.265, 0.116] | 0.019 [-0.006, 0.044] | -0.024 [-0.044, -0.003] | 0.179 [0.005, 0.352] | -0.001 [-0.154, 0.151] |
| Sex X Age | 0.009 [-0.153, 0.170] | -0.032 [-0.149, 0.086] | -0.027 [-0.140, 0.087] | 0.093 [-0.017, 0.203] | -0.132 [-0.270, 0.005] | -0.046 [-0.185, 0.094] | -0.042 [-0.167, 0.083] | -0.114 [-0.267, 0.040] | -0.018 [-0.037, 0.002] | -0.005 [-0.021, 0.011] | 0.003 [-0.136, 0.142] | 0.116 [-0.001, 0.234] |
| Year X Support | 0.314 [-0.178, 0.805] | 0.463 [0.041, 0.886] | -0.515 [-0.857, -0.174] | -0.690 [-1.037, -0.342] | 1.052 [0.672, 1.432] | 0.777 [0.390, 1.164] | -1.353 [-1.748, -0.958] | -1.335 [-1.796, -0.875] | -0.114 [-0.173, -0.054] | 0.212 [0.163, 0.261] | -0.611 [-1.000, -0.222] | -0.159 [-0.508, 0.190] |
| Year X Sex X Age | 0.048 [-0.239, 0.336] | -0.038 [-0.275, 0.198] | 0.185 [-0.035, 0.404] | -0.105 [-0.311, 0.101] | 0.250 [0.012, 0.488] | -0.049 [-0.297, 0.200] | 0.021 [-0.211, 0.253] | 0.254 [-0.024, 0.532] | 0.022 [-0.013, 0.058] | 0.001 [-0.028, 0.031] | 0.009 [-0.233, 0.251] | -0.162 [-0.385, 0.061] |

**Supplementary Table 10**. Estimates and 95% confidence intervals from multilevel modeling testing social support as an effect modifier of temporal changes in well-being in Syria.

| | **Problems** | **Pain** | **Rested** | **Health Care** | **Cannot Afford Food** | **Cannot Afford Shelter** | **Life Sat.** | **Hope** | **Pos. Emot.** | **Neg. Emot.** | **Respect** | **Freedom** |
|---|---|---|---|---|---|---|---|---|---|---|---|---|
| **Social Support as an Effect Modifier** | | | | | | | | | | | | |
| N | 6743 | 7691 | 8094 | 7463 | 7108 | 8174 | 8634 | 8018 | 8736 | 8734 | 7889 | 8136 |
| Intercept | -1.374 | -0.306 | 0.416 | 0.097 | -0.798 | -1.433 | 4.507 | 5.302 | 0.608 | 0.435 | 1.763 | 0.699 |
| | [-1.662; -1.087] | [-0.511; -0.100] | [0.233; 0.598] | [-0.108; 0.302] | [-1.014; -0.583] | [-1.655; -1.211] | [4.303; 4.711] | [5.056; 5.549] | [0.576; 0.640] | [0.407; 0.462] | [1.518; 2.008] | [0.515; 0.882] |
| Year | -0.482 | -1.946 | -0.433 | -0.873 | 0.791 | 0.790 | -1.639 | -0.355 | -0.191 | 0.228 | -1.717 | -1.145 |
| | [-0.903; -0.061] | [-2.319; -1.573] | [-0.741; -0.126] | [-1.177; -0.569] | [0.460; 1.123] | [0.445; 1.134] | [-1.984; -1.295] | [-0.769; 0.060] | [-0.244; -0.137] | [0.184; 0.272] | [-2.073; -1.361] | [-1.458; -0.832] |
| Sex | 0.305 | 0.238 | 0.047 | -0.115 | -0.342 | -0.306 | 0.314 | 0.376 | -0.001 | 0.012 | -0.336 | -0.060 |
| | [0.062; 0.547] | [0.062; 0.415] | [-0.117; 0.212] | [-0.278; 0.047] | [-0.549; -0.135] | [-0.512; -0.101] | [0.130; 0.498] | [0.154; 0.597] | [-0.030; 0.028] | [-0.012; 0.035] | [-0.547; -0.124] | [-0.229; 0.110] |
| Age | -0.036 | 0.154 | 0.037 | -0.041 | 0.133 | 0.047 | 0.013 | 0.017 | -0.013 | 0.016 | -0.113 | -0.008 |
| | [-0.153; 0.081] | [0.070; 0.237] | [-0.042; 0.116] | [-0.120; 0.037] | [0.038; 0.228] | [-0.045; 0.140] | [-0.076; 0.102] | [-0.089; 0.122] | [-0.027; 0.000] | [0.005; 0.027] | [-0.214; -0.011] | [-0.089; 0.073] |
| Support | -0.391 | -0.414 | 0.379 | 0.661 | -0.867 | -0.379 | 0.898 | 1.121 | 0.063 | -0.162 | 0.502 | 0.215 |
| | [-0.648; -0.133] | [-0.615; -0.213] | [0.196; 0.561] | [0.479; 0.843] | [-1.080; -0.654] | [-0.602; -0.156] | [0.689; 1.106] | [0.867; 1.375] | [0.031; 0.095] | [-0.189; -0.136] | [0.275; 0.729] | [0.025; 0.406] |
| Year X Sex | -0.723 | -0.436 | -0.072 | 0.346 | 0.311 | 0.451 | -0.495 | -0.241 | -0.005 | -0.014 | 0.683 | -0.171 |
| | [-1.198; -0.248] | [-0.833; -0.040] | [-0.398; 0.254] | [0.021; 0.670] | [-0.063; 0.685] | [0.079; 0.823] | [-0.854; -0.135] | [-0.668; 0.186] | [-0.061; 0.051] | [-0.060; 0.032] | [0.302; 1.064] | [-0.502; 0.160] |
| Year X Age | 0.186 | 0.125 | -0.163 | 0.091 | -0.269 | -0.034 | -0.040 | -0.050 | 0.024 | -0.029 | 0.233 | -0.001 |
| | [-0.024; 0.396] | [-0.044; 0.294] | [-0.317; -0.009] | [-0.059; 0.242] | [-0.439; -0.100] | [-0.200; 0.132] | [-0.209; 0.128] | [-0.247; 0.147] | [-0.002; 0.050] | [-0.051; -0.008] | [0.053; 0.413] | [-0.154; 0.151] |
| Sex X Age | 0.002 | -0.062 | -0.027 | 0.101 | -0.166 | -0.046 | -0.036 | -0.108 | -0.014 | -0.007 | -0.007 | 0.116 |
| | [-0.163; 0.168] | [-0.181; 0.057] | [-0.140; 0.087] | [-0.011; 0.212] | [-0.310; -0.023] | [-0.185; 0.094] | [-0.163; 0.090] | [-0.260; 0.044] | [-0.034; 0.006] | [-0.023; 0.009] | [-0.148; 0.135] | [-0.001; 0.234] |
| Year X Support | 0.316 | 0.595 | -0.515 | -0.831 | 1.437 | 0.777 | -1.426 | -1.658 | -0.127 | 0.225 | -0.640 | -0.159 |
| | [-0.168; 0.800] | [0.181; 1.008] | [-0.857; -0.174] | [-1.171; -0.491] | [1.057; 1.816] | [0.390; 1.164] | [-1.807; -1.044] | [-2.116; -1.200] | [-0.186; -0.068] | [0.177; 0.274] | [-1.035; -0.246] | [-0.508; 0.190] |
| Year X Sex X Age | 0.067 | 0.043 | 0.185 | -0.121 | 0.307 | -0.049 | 0.006 | 0.249 | 0.014 | 0.010 | 0.044 | -0.162 |
| | [-0.238; 0.373] | [-0.204; 0.289] | [-0.035; 0.404] | [-0.337; 0.095] | [0.051; 0.562] | [-0.297; 0.200] | [-0.236; 0.247] | [-0.037; 0.536] | [-0.024; 0.051] | [-0.021; 0.041] | [-0.210; 0.298] | [-0.385; 0.061] |

**Supplementary Table 11.** Descriptive statistics of well-being measures for Syria and the World with Syria's rank of well-being among 163 countries.

| Variables | Measure | **2006** | **2007** | **2008** | **2009** | **2010** | **2011** | **2012** | **2013** | **2014** | **2015** | **2016** |
|---|---|---|---|---|---|---|---|---|---|---|---|---|
| Number of Participants | | 129037 | 104755 | 133501 | 139049 | 155861 | 198400 | 231460 | 138171 | 190344 | 152256 | 149724 |
| Number of Countries | | 123 | 96 | 121 | 115 | 124 | 148 | 142 | 136 | 148 | 141 | 141 |
| **Physical Well-being** | | | | | | | | | | | | |
| Health Problems | World | 24.8% | 24.5% | 24.8% | 25.8% | 25.2% | 23.9% | 23.6% | 24.0% | 24.1% | 24.3% | 25.4% |
| | Syria | | | 25.3% | 23.2% | 12.0% | 12.0% | 11.0% | 15.0% | | 17.0% | |
| | Syria rank | | | 67 | 44.5 | **2** | **3** | 1.5 | 8 | | 21.5 | |
| Physical Pain | World | 27.0% | 25.5% | 26.9% | 27.3% | 26.9% | 28.4% | 29.4% | 30.5% | 31.1% | 31.0% | 33.0% |
| | Syria | | | 38.0% | 33.0% | 16.2% | 26.3% | 16.3% | 14.4% | | 15.0% | |
| | Syria rank | | | 107 | 91.5 | 10 | 62.5 | 7 | **2** | | **1.5** | |
| Well-Rested | World | 65.2% | 66.8% | 66.9% | 68.3% | 69.6% | 67.8% | 67.6% | 66.2% | 66.3% | 66.8% | 66.7% |
| | Syria | | | 56.6% | 65.7% | 60.2% | 66.7% | 64.3% | 37.8% | | 35.8% | |
| | Syria rank | | | 102 | 77 | 98.5 | 82.5 | 90.5 | **136*** | | **141*** | |
| Satisfactory Access to Health Care | World | 56.5% | 56.5% | 56.5% | 56.5% | 56.5% | 56.5% | 56.5% | 56.5% | 56.5% | 56.5% | 56.5% |
| | Syria | | | 54.1% | 68.4% | 77.1% | 38.9% | 31.9% | 39.8% | | 39.2% | |
| | Syria rank | | | 71 | 32 | 22 | 113 | 119 | 101 | | 110.5 | |
| Cannot Afford Food | World | 29.6% | 30.7% | 32.1% | 30.8% | 30.5% | 32.2% | 31.7% | 33.7% | 33.7% | 33.8% | 36.0% |
| | Syria | | | 16.5% | 16.0% | | 37.1% | 41.9% | 57.0% | | 48.1% | |
| | Syria rank | | | 36 | 28.5 | | 91 | 98 | 112 | | 98 | |
| Cannot Afford Shelter | World | 19.7% | 21.0% | 20.0% | 20.3% | 21.2% | 21.4% | 22.9% | 23.4% | 23.8% | 23.8% | 26.8% |
| | Syria | | | 13.4% | 20.0% | 25.5% | 19.6% | 29.9% | 44.6% | | 34.8% | |
| | Syria rank | | | 48 | 62 | 78.5 | 73 | 98 | 119 | | 106 | |
| **Mental Well-being** | | | | | | | | | | | | |
| Average Life Satisfaction | World | 5.4 | 5.5 | 5.5 | 5.5 | 5.5 | 5.4 | 5.5 | 5.4 | 5.4 | 5.4 | 5.4 |
| (0 to 10) | Syria | | | 5.3 | 5.0 | 4.5 | 4.0 | 3.2 | 2.7 | | 3.5 | |
| | Syria rank | | | 56 | 76 | 97.5 | 133.5 | **141.5*** | **135*** | | **138** | |
| Average Hope | World | 6.8 | 6.8 | 6.7 | 6.7 | 6.7 | 6.7 | 6.7 | 6.7 | 6.8 | 6.8 | 6.8 |
| (0 to 10) | Syria | | | 6.3 | 6.3 | 5.7 | 5.2 | 5.4 | 5.6 | | 4.9 | |
| | Syria rank | | | 74 | 71.5 | 104 | 133.5 | 123 | 112.5 | | **133** | |
| Positive Emotions | World | 71.5% | 71.9% | 70.7% | 70.6% | 70.9% | 70.4% | 70.9% | 70.9% | 70.9% | 71.0% | 71.2% |
| | Syria | | | 62.6% | 58.4% | 53.5% | 65.2% | 48.4% | 38.8% | | 37.0% | |
| | Syria rank | | | 88 | 94 | 112 | 98.5 | **140** | **136*** | | **141*** | |
| Negative Emotions | World | 26.6% | 24.9% | 25.0% | 25.1% | 25.0% | 26.0% | 27.3% | 28.7% | 28.6% | 29.4% | 30.1% |
| | Syria | | | 37.7% | 32.5% | 26.7% | 51.2% | 66.6% | 64.8% | | 62.5% | |
| | Syria rank | | | 110 | 98 | 76 | **147** | **142*** | **136*** | | **141*** | |
| **Social Well-being** | | | | | | | | | | | | |
| Social Support | World | 84.8% | 80.9% | 79.0% | 82.0% | 83.2% | 80.2% | 80.7% | 80.5% | 80.4% | 79.6% | 81.0% |
| | Syria | | | 71.7% | 84.0% | 92.8% | 57.9% | 58.7% | 58.8% | | 46.8% | |
| | Syria rank | | | 86.5 | 54.5 | 20 | 137 | 135 | 129 | | **140** | |
| Respect | World | 85.2% | 84.4% | 85.0% | 87.0% | 88.0% | 86.9% | 87.1% | 86.2% | 86.0% | 87.0% | 87.9% |

| | | | | | | | | | | | | |
|---|---|---|---|---|---|---|---|---|---|---|---|---|
| | Syria | | | 80.8% | 91.9% | 87.8% | 78.8% | 67.9% | 61.4% | | 57.6% | |
| | Syria rank | | | 81 | 37 | 77 | 130 | **138** | **134** | | **141***  | |
| Freedom | World | 73.5% | 70.7% | 69.2% | 68.9% | 70.9% | 73.4% | 71.2% | 72.8% | 73.7% | 74.9% | 76.4% |
| | Syria | | | 65.6% | 75.0% | 65.2% | 52.8% | 47.1% | 46.0% | | 45.3% | |
| | Syria rank | | | 62 | 44 | 83 | 132 | 130 | 128 | | **134** | |

Note. Bolded numbers highlight years when Syria ranked in the top or bottom 5 in a specific wave. Asterisks highlight years when Syria ranked the lowest in the sample.

**Supplementary Table 12.** Estimates and 95% confidence intervals of physical, mental, and social well-being in WHO regions and Syria in 2006-2016.

| | **Health Problems** | **Pain** | **Rested** | **Health Care** | **Cannot Afford Food** | **Cannot Afford Shelter** | **Life Sat.** | **Hope** | **Pos. Emot.** | **Neg. Emot.** | **Support** | **Respect** | **Freedom** |
|---|---|---|---|---|---|---|---|---|---|---|---|---|---|
| Number of countries | 162 | 163 | 163 | 163 | 162 | 162 | 163 | 156 | 163 | 163 | 162 | 163 | 163 |
| Number of observations | 1419 | 1422 | 1422 | 1422 | 1407 | 1407 | 1424 | 1346 | 1428 | 1424 | 1420 | 1425 | 1397 |
| Intercept | 0.203 | 0.330 | 0.673 | 0.641 | 0.166 | 0.155 | 5.095 | 6.226 | 0.643 | 0.315 | 0.834 | 0.903 | 0.701 |
| | [0.096; 0.309] | [0.206; 0.454] | [0.528; 0.818] | [0.309; 0.974] | [-0.113; 0.446] | [-0.096; 0.406] | [3.335; 6.855] | [4.615; 7.838] | [0.467; 0.818] | [0.192; 0.439] | [0.629; 1.040] | [0.757; 1.049] | [0.419; 0.983] |
| Year | -0.084 | -0.228 | -0.268 | -0.317* | 0.409 | 0.253 | -2.375 | -1.331 | -0.274 | 0.386 | -0.360 | -0.337 | -0.298 |
| | [-0.194; 0.026] | [-0.348; -0.108] | [-0.384; -0.152] | [-0.515; -0.118] | [0.216; 0.602] | [0.064; 0.441] | [-3.403; -1.348] | [-2.518; -0.145] | [-0.388; -0.159] | [0.267; 0.506] | [-0.490; -0.231] | [-0.435; -0.239] | [-0.498; -0.098] |
| Africa | 0.058 | -0.035 | -0.009 | -0.250 | 0.387 | 0.138 | -0.738 | 0.382 | 0.030 | -0.102 | -0.121 | -0.083 | -0.052 |
| | [-0.050; 0.166] | [-0.160; 0.090] | [-0.155; 0.138] | [-0.587; 0.086] | [0.104; 0.669] | [-0.116; 0.393] | [-2.521; 1.045] | [-1.251; 2.016] | [-0.148; 0.207] | [-0.227; 0.024] | [-0.329; 0.087] | [-0.231; 0.064] | [-0.337; 0.233] |
| America | 0.022 | -0.048 | 0.043 | -0.039 | 0.170 | 0.027 | 0.980 | 1.029 | 0.157 | -0.042 | 0.033 | 0.002 | 0.041 |
| | [-0.087; 0.130] | [-0.174; 0.078] | [-0.104; 0.190] | [-0.377; 0.300] | [-0.114; 0.455] | [-0.228; 0.283] | [-0.811; 2.771] | [-0.613; 2.671] | [-0.021; 0.336] | [-0.167; 0.084] | [-0.176; 0.242] | [-0.147; 0.150] | [-0.246; 0.328] |
| SE. Asia | 0.053 | -0.082 | 0.040 | 0.033 | 0.059 | 0.015 | -0.199 | 0.270 | 0.112 | -0.100 | -0.088 | -0.189 | 0.005 |
| | [-0.060; 0.167] | [-0.214; 0.049] | [-0.114; 0.193] | [-0.320; 0.386] | [-0.238; 0.355] | [-0.252; 0.282] | [-2.068; 1.669] | [-1.442; 1.981] | [-0.074; 0.298] | [-0.231; 0.031] | [-0.306; 0.130] | [-0.344; -0.034] | [-0.295; 0.304] |
| Europe | 0.058 | -0.075 | -0.049 | -0.017 | 0.025 | 0.004 | 0.798 | 0.444 | 0.040 | -0.058 | 0.037 | -0.023 | 0.009 |
| | [-0.050; 0.165] | [-0.199; 0.050] | [-0.195; 0.097] | [-0.353; 0.318] | [-0.257; 0.307] | [-0.250; 0.257] | [-0.979; 2.575] | [-1.183; 2.071] | [-0.137; 0.217] | [-0.183; 0.067] | [-0.170; 0.244] | [-0.170; 0.124] | [-0.276; 0.293] |
| E. Mediterranean | 0.036 | 0.004 | 0.011 | -0.089 | 0.068 | 0.056 | 0.304 | 0.495 | 0.013 | -0.007 | -0.052 | -0.036 | -0.034 |
| | [-0.073; 0.146] | [-0.123; 0.131] | [-0.137; 0.160] | [-0.430; 0.251] | [-0.219; 0.354] | [-0.202; 0.313] | [-1.500; 2.108] | [-1.159; 2.148] | [-0.167; 0.193] | [-0.134; 0.120] | [-0.263; 0.159] | [-0.186; 0.113] | [-0.323; 0.255] |
| W. Pacific | 0.017 | -0.120 | 0.048 | 0.065 | 0.078 | 0.042 | 0.379 | 0.438 | 0.095 | -0.073 | -0.022 | -0.099 | 0.096 |
| | [-0.093; 0.127] | [-0.248; 0.007] | [-0.101; 0.197] | [-0.278; 0.408] | [-0.210; 0.366] | [-0.217; 0.301] | [-1.437; 2.196] | [-1.228; 2.103] | [-0.086; 0.276] | [-0.200; 0.054] | [-0.234; 0.190] | [-0.250; 0.051] | [-0.195; 0.386] |
| Year X Africa | 0.111 | 0.307 | 0.263 | 0.333 | -0.376 | -0.155 | 2.240 | 1.402 | 0.282 | -0.311 | 0.362 | 0.347 | 0.348 |
| | [-0.000; 0.222] | [0.186; 0.428] | [0.146; 0.380] | [0.132; 0.534] | [-0.572; -0.181] | [-0.345; 0.036] | [1.201; 3.279] | [0.201; 2.602] | [0.167; 0.398] | [-0.432; -0.190] | [0.231; 0.493] | [0.248; 0.445] | [0.146; 0.551] |
| Year X America | 0.073 | 0.276 | 0.259 | 0.264 | -0.367 | -0.200 | 2.483 | 1.623 | 0.280 | -0.343 | 0.341 | 0.348 | 0.347 |
| | [-0.039; 0.184] | [0.154; 0.397] | [0.142; 0.376] | [0.062; 0.465] | [-0.563; -0.170] | [-0.391; -0.009] | [1.441; 3.525] | [0.418; 2.828] | [0.164; 0.396] | [-0.464; -0.222] | [0.210; 0.472] | [0.249; 0.447] | [0.144; 0.549] |
| Year X SE. Asia | 0.088 | 0.310 | 0.277 | 0.380 | -0.305 | -0.160 | 2.415 | 1.429 | 0.287 | -0.345 | 0.377 | 0.412 | 0.413 |
| | [-0.027; 0.203] | [0.185; 0.436] | [0.157; 0.398] | [0.170; 0.590] | [-0.509; -0.101] | [-0.359; 0.039] | [1.336; 3.495] | [0.182; 2.675] | [0.167; 0.407] | [-0.470; -0.220] | [0.242; 0.513] | [0.310; 0.513] | [0.203; 0.624] |
| Year X Europe | 0.071 | 0.243 | 0.286 | 0.330 | -0.407 | -0.254 | 2.446 | 1.275 | 0.284 | -0.377 | 0.357 | 0.352 | 0.331 |
| | [-0.040; 0.182] | [0.123; 0.364] | [0.170; 0.403] | [0.130; 0.530] | [-0.601; -0.212] | [-0.444; -0.065] | [1.411; 3.481] | [0.080; 2.469] | [0.169; 0.399] | [-0.497; -0.256] | [0.227; 0.488] | [0.253; 0.450] | [0.129; 0.532] |
| Year X E. Mediterranean | 0.053 | 0.277 | 0.246 | 0.305 | -0.312 | -0.203 | 2.182 | 1.256 | 0.259 | -0.346 | 0.315 | 0.364 | 0.319 |

| | | | | | | | | | | | | | |
|---|---|---|---|---|---|---|---|---|---|---|---|---|---|
| | [-0.059; 0.166] | [0.155; 0.400] | [0.128; 0.364] | [0.102; 0.509] | [-0.510; -0.114] | [-0.396; -0.010] | [1.132; 3.232] | [0.042; 2.470] | [0.142; 0.376] | [-0.469; -0.224] | [0.183; 0.447] | [0.265; 0.464] | [0.114; 0.524] |
| Year X W. Pacific | 0.092 | 0.266 | 0.287 | 0.329 | -0.374 | -0.201 | 2.518 | 1.506 | 0.293 | -0.364 | 0.361 | 0.368 | 0.325 |
| | [-0.021; 0.204] | [0.143; 0.388] | [0.168; 0.405] | [0.126; 0.533] | [-0.572; -0.176] | [-0.395; -0.008] | [1.467; 3.569] | [0.290; 2.722] | [0.176; 0.410] | [-0.486; -0.242] | [0.229; 0.493] | [0.269; 0.468] | [0.120; 0.530] |

**Supplementary Table 13.** Estimated changes and 95% confidence intervals of physical, mental, and social well-being in WHO regions and Syria in 2006-2016.

| | | Syria | E. Mediterranean | Africa | America | SE. Asia | Europe | W. Pacific |
|---|---|---|---|---|---|---|---|---|
| Health Problems | | -0.084 | **-0.031** | 0.027 | -0.011 | 0.004 | -0.013 | -0.008 |
| | Lower CI | -0.194 | -0.054 | 0.011 | -0.029 | -0.031 | -0.026 | -0.016 |
| | Upper CI | 0.026 | -0.008 | 0.043 | 0.007 | 0.038 | 0.000 | 0.032 |
| Pain | | **-0.228** | **0.049** | **0.081** | **0.048** | **0.082** | **0.015** | **0.038** |
| | Lower CI | -0.349 | 0.024 | 0.064 | 0.029 | 0.045 | 0.001 | 0.013 |
| | Upper CI | -0.108 | 0.074 | 0.098 | 0.067 | 0.119 | 0.029 | 0.063 |
| Rested | | **-0.268** | -0.022 | -0.005 | -0.009 | 0.009 | 0.018 | 0.019 |
| | Lower CI | -0.384 | -0.045 | -0.021 | -0.023 | -0.025 | 0.006 | -0.004 |
| | Upper CI | -0.152 | 0.001 | 0.011 | 0.009 | 0.043 | 0.030 | 0.042 |
| Health Care | | **-0.317** | -0.012 | 0.017 | **-0.053** | 0.063 | 0.015 | 0.012 |
| | Lower CI | -0.516 | -0.057 | -0.014 | -0.088 | -0.005 | -0.010 | -0.034 |
| | Upper CI | -0.117 | 0.033 | 0.048 | -0.005 | 0.131 | 0.040 | 0.058 |
| Cannot Afford Food | | **0.409** | **0.097** | **0.015** | **0.042** | **0.104** | 0.003 | 0.035 |
| | Lower CI | 0.214 | 0.055 | 0.005 | 0.008 | 0.039 | -0.021 | -0.009 |
| | Upper CI | 0.604 | 0.139 | 0.063 | 0.075 | 0.169 | 0.027 | 0.079 |
| Cannot Afford Shelter | | **0.253** | **0.050** | **0.099** | **0.053** | **0.093** | -0.001 | **0.052** |
| | Lower CI | 0.063 | 0.008 | 0.070 | 0.020 | 0.030 | -0.024 | 0.009 |
| | Upper CI | 0.442 | 0.092 | 0.128 | 0.086 | 0.156 | 0.022 | 0.095 |
| Life Satisfaction | | **-2.375** | -0.193 | -0.132 | 0.108 | 0.040 | 0.068 | 0.143 |
| | Lower CI | -3.407 | -0.410 | -0.285 | -0.063 | -0.292 | -0.052 | -0.079 |
| | Upper CI | -1.344 | 0.024 | 0.021 | 0.279 | 0.372 | 0.188 | 0.365 |
| Hope | | **-1.331** | -0.077 | 0.060 | **0.291** | 0.098 | -0.057 | 0.175 |
| | Lower CI | -2.526 | -0.337 | -0.124 | 0.079 | -0.288 | -0.198 | -0.093 |
| | Upper CI | -0.136 | 0.183 | 0.244 | 0.503 | 0.484 | 0.084 | 0.443 |
| Positive Emotion | | **-0.274** | -0.015 | 0.010 | 0.006 | 0.013 | 0.010 | 0.019 |
| | Lower CI | -0.390 | -0.147 | -0.007 | -0.012 | -0.023 | -0.003 | -0.005 |
| | Upper CI | -0.158 | 0.117 | 0.023 | 0.024 | 0.049 | 0.023 | 0.043 |
| Negative Emotion | | **0.386** | **0.040** | **0.075** | **0.043** | **0.041** | 0.010 | 0.022 |
| | Lower CI | 0.267 | 0.015 | 0.058 | 0.024 | 0.004 | -0.004 | -0.003 |
| | Upper CI | 0.506 | 0.065 | 0.092 | 0.062 | 0.078 | 0.024 | 0.047 |
| Support | | **-0.360** | **-0.046** | 0.000 | -0.019 | 0.019 | 0.003 | 0.001 |
| | Lower CI | -0.496 | -0.075 | -0.020 | -0.041 | -0.024 | -0.012 | -0.028 |
| | Upper CI | -0.225 | -0.017 | 0.020 | 0.003 | 0.062 | 0.018 | 0.030 |
| Respect | | **-0.298** | **0.021** | **0.051** | **0.049** | **0.116** | **0.032** | **0.027** |
| | Lower CI | -0.499 | 0.002 | 0.038 | 0.035 | 0.088 | 0.022 | 0.008 |
| | Upper CI | -0.097 | 0.040 | 0.064 | 0.063 | 0.144 | 0.042 | 0.046 |
| Freedom | | **-0.337** | 0.027 | 0.011 | 0.011 | 0.075 | 0.014 | 0.031 |
| | Lower CI | -0.436 | -0.017 | -0.019 | -0.023 | 0.010 | -0.051 | -0.014 |
| | Upper CI | -0.238 | 0.071 | 0.041 | 0.045 | 0.140 | 0.079 | 0.076 |

Note. The 95%CI of bolded estimates exclude 0.

**Supplementary Table 14**. Estimates and 95% confidence intervals of physical, mental, and social well-being in Syria and countries that experiences war, protests, or disasters in 2006-2016.

| | **Health Problems** | **Pain** | **Rested** | **Health Care** | **Cannot Afford Food** | **Cannot Afford Shelter** | **Life Sat.** | **Hope** | **Pos. Emot.** | **Neg. Emot.** | **Support** | **Respect** | **Freedom** |
|---|---|---|---|---|---|---|---|---|---|---|---|---|---|
| Number of countries | 34 | 34 | 34 | 34 | 34 | 34 | 34 | 32 | 34 | 34 | 34 | 34 | 34 |
| Number of observations | 367 | 368 | 368 | 352 | 355 | 357 | 362 | 345 | 368 | 368 | 367 | 368 | 354 |
| Intercept | 0.167 | 0.232 | 0.558 | 0.506 | 0.341 | 0.263 | 4.077 | 5.656 | 0.525 | 0.481 | 0.680 | 0.759 | 0.573 |
| | [0.051; 0.283] | [0.069; 0.395] | [0.430; 0.687] | [0.089; 0.922] | [-0.067; 0.750] | [-0.008; 0.535] | [2.010; 6.144] | [3.985; 7.327] | [0.349; 0.702] | [0.332; 0.630] | [0.449; 0.911] | [0.606; 0.912] | [0.318; 0.828] |
| Year to events | -0.084 | -0.228 | -0.268 | -0.317 | 0.409 | 0.253 | -2.375 | -1.331 | -0.274 | 0.386 | -0.360 | -0.337 | -0.298 |
| | [-0.179; 0.012] | [-0.354; -0.103] | [-0.394; -0.141] | [-0.525; -0.109] | [0.195; 0.623] | [0.066; 0.439] | [-3.454; -1.297] | [-2.580; -0.082] | [-0.384; -0.163] | [0.255; 0.518] | [-0.486; -0.235] | [-0.446; -0.228] | [-0.490; -0.106] |
| War-torn countries | 0.074 | 0.079 | 0.083 | 0.058 | 0.059 | 0.016 | 1.068 | 1.094 | 0.125 | -0.171 | 0.107 | 0.073 | 0.125 |
| | [-0.046; 0.194] | [-0.089; 0.247] | [-0.050; 0.217] | [-0.367; 0.483] | [-0.359; 0.477] | [-0.262; 0.295] | [-1.047; 3.184] | [-0.627; 2.814] | [-0.056; 0.307] | [-0.325; -0.017] | [-0.131; 0.344] | [-0.084; 0.231] | [-0.138; 0.388] |
| Countries with major protests | 0.085 | 0.066 | 0.125 | 0.028 | 0.009 | -0.019 | 1.189 | 1.081 | 0.180 | -0.211 | 0.104 | 0.087 | 0.141 |
| | [-0.034; 0.204] | [-0.101; 0.234] | [-0.008; 0.257] | [-0.397; 0.452] | [-0.408; 0.425] | [-0.297; 0.258] | [-0.918; 3.296] | [-0.628; 2.790] | [-0.000; 0.361] | [-0.364; -0.059] | [-0.132; 0.340] | [-0.070; 0.243] | [-0.120; 0.403] |
| Countries with natural disasters | 0.090 | 0.072 | 0.112 | 0.005 | 0.029 | 0.006 | 1.040 | 1.010 | 0.169 | -0.205 | 0.089 | 0.074 | 0.116 |
| | [-0.028; 0.208] | [-0.094; 0.238] | [-0.019; 0.243] | [-0.418; 0.428] | [-0.387; 0.444] | [-0.271; 0.282] | [-1.061; 3.141] | [-0.692; 2.711] | [-0.010; 0.349] | [-0.356; -0.053] | [-0.147; 0.324] | [-0.081; 0.230] | [-0.144; 0.376] |
| Year X War-torn | 0.047 | 0.242 | 0.268 | 0.360 | -0.364 | -0.246 | 2.164 | 1.309 | 0.246 | -0.375 | 0.393 | 0.373 | 0.322 |
| | [-0.052; 0.146] | [0.112; 0.372] | [0.137; 0.399] | [0.144; 0.575] | [-0.586; -0.142] | [-0.440; -0.052] | [1.045; 3.284] | [0.008; 2.610] | [0.132; 0.360] | [-0.510; -0.239] | [0.264; 0.522] | [0.260; 0.485] | [0.124; 0.520] |
| Year X Protests | 0.078 | 0.270 | 0.267 | 0.287 | -0.373 | -0.205 | 2.442 | 1.371 | 0.281 | -0.352 | 0.348 | 0.356 | 0.323 |
| | [-0.020; 0.176] | [0.141; 0.398] | [0.138; 0.397] | [0.072; 0.501] | [-0.594; -0.153] | [-0.398; -0.013] | [1.332; 3.553] | [0.085; 2.658] | [0.168; 0.394] | [-0.487; -0.217] | [0.220; 0.477] | [0.244; 0.468] | [0.126; 0.520] |
| Year X Disasters | 0.076 | 0.270 | 0.278 | 0.350 | -0.389 | -0.222 | 2.405 | 1.253 | 0.265 | -0.356 | 0.356 | 0.360 | 0.350 |
| | [-0.021; 0.173] | [0.142; 0.397] | [0.150; 0.407] | [0.139; 0.562] | [-0.607; -0.171] | [-0.412; -0.031] | [1.307; 3.504] | [-0.020; 2.526] | [0.153; 0.377] | [-0.489; -0.222] | [0.229; 0.484] | [0.249; 0.471] | [0.155; 0.545] |

**Supplementary Table 15**. Missing Data Pattern. Percentages represent the proportion of completed responses.

| Wave | 2008 | 2009.1 | 2009.2 | 2010.1 | 2010.2 | 2011.1 | 2011.2 | 2012.1 | 2012.2 | 2013 | 2015 |
|---|---|---|---|---|---|---|---|---|---|---|---|
| Sample Size | 1209 | 1082 | 1018 | 1029 | 1006 | 1011 | 1030 | 1025 | 1018 | 1022 | 1002 |
| Sex | 100.0% | 100.0% | 100.0% | 100.0% | 100.0% | 100.0% | 100.0% | 100.0% | 100.0% | 100.0% | 100.0% |
| Age | 99.9% | 100.0% | 99.8% | 99.9% | 100.0% | 100.0% | 100.0% | 100.0% | 99.9% | 100.0% | 100.0% |
| Health Problems | 99.3% | - | 99.5% | 99.7% | - | 100.0% | 100.0% | 100.0% | - | 100.0% | 100.0% |
| Pain | 99.4% | 99.9% | 99.8% | 99.4% | - | 99.3% | - | 100.0% | 96.0% | 97.2% | 100.0% |
| Well-rested | 98.3% | 99.6% | 98.8% | 98.1% | - | 98.9% | 98.3% | 95.0% | 75.0% | 81.9% | 81.8% |
| Health Care | 97.9% | 98.2% | - | 95.7% | - | 95.6% | 94.6% | - | 94.0% | 92.4% | 96.7% |
| Cannot Afford Food | 98.7% | 99.9% | - | - | - | 97.6% | 97.0% | 89.1% | 84.0% | 79.0% | 81.7% |
| Cannot Afford Shelter | 98.8% | 99.9% | 99.6% | 98.0% | - | 96.6% | 97.5% | 89.0% | 84.0% | 83.2% | 89.0% |
| Life Satisfaction | 99.8% | 100.0% | 99.7% | 98.7% | 99.0% | 100.0% | 99.7% | 98.2% | 97.5% | 96.7% | 96.1% |
| Hope | 83.5% | 92.2% | 77.3% | 82.9% | 82.7% | 99.0% | 98.8% | 96.9% | 91.3% | 90.8% | 91.6% |
| Positive Emotion | 99.7% | 99.8% | 97.8% | 100.0% | 98.1% | 100.0% | 100.0% | 99.6% | 99.6% | 100.0% | 100.0% |
| Negative Emotion | 99.7% | 99.9% | 99.6% | 99.8% | - | 100.0% | 100.0% | 100.0% | 99.0% | 100.0% | 100.0% |
| Support | 98.4% | 99.4% | - | 96.5% | - | 95.2% | 94.8% | 93.6% | 91.0% | 85.7% | 79.5% |
| Respect | 97.8% | 99.4% | 99.1% | 97.8% | 98.4% | 99.4% | 98.9% | 84.0% | 86.1% | 84.0% | 84.7% |
| Freedom | 95.2% | 97.1% | 94.9% | 92.4% | 93.3% | 83.1% | 95.2% | 81.0% | 90.0% | 87.4% | 85.2% |
| **Exposure Status** | | | | | | | | | | | |
| Internally Displaced (Self) | - | - | - | - | - | - | - | - | - | 91.2% | 90.9% |
| Internally Displaced to the Same Governorate (Family) | - | - | - | - | - | - | - | - | - | 88.3% | 91.7% |
| Internally Displaced to a Different Governorate (Family) | - | - | - | - | - | - | - | - | - | 88.9% | 91.7% |
| Internally Displaced to Somewhere outside Syria (Family) | - | - | - | - | - | - | - | - | - | 88.5% | 91.7% |
| Died (Family) | - | - | - | - | - | - | - | - | - | 84.2% | 87.8% |
| Injured (Family) | - | - | - | - | - | - | - | - | - | 85.6% | 88.3% |
| Household Lost Main Source of Income | - | - | - | - | - | - | - | - | - | 98.1% | 86.9% |

Notes. ‘–‘ indicates that the measure was not included in a specific survey. In 2009, 2010, 2011, and 2012, Gallup conducted 2 surveys in the same calendar year, and each survey can include different items. The suffices, ‘.1’ or ‘.2’, refers to the first or the second survey in the same year.
