# Supplementary material for: The impact of the Syrian conflict on population well-being
Source: Nat Commun. 2020 Aug 6;11:3899. doi: 10.1038/s41467-020-17369-0 (PMC7411009; doi:10.1038/s41467-020-17369-0)
Supplement: Supplementary file 3 — Reporting Summary [file 41467_2020_17369_MOESM3_ESM.pdf]

nature research

Corresponding author(s): DBPR NCOMMS-19-10187A

Last updated by author(s): Jun 2, 2020

Reporting Summary

Nature Research wishes to improve the reproducibility of the work that we publish. This form provides structure for consistency and transparency in reporting. For further information on Nature Research policies, see Authors & Referees and the Editorial Policy Checklist.

Statistics

For all statistical analyses, confirm that the following items are present in the figure legend, table legend, main text, or Methods section.

n/a	Confirmed	
☐	☒	The exact sample size (*n*) for each experimental group/condition, given as a discrete number and unit of measurement
☐	☒	A statement on whether measurements were taken from distinct samples or whether the same sample was measured repeatedly
☐	☒	The statistical test(s) used AND whether they are one- or two-sided<br>*Only common tests should be described solely by name; describe more complex techniques in the Methods section.*
☐	☒	A description of all covariates tested
☐	☒	A description of any assumptions or corrections, such as tests of normality and adjustment for multiple comparisons
☐	☒	A full description of the statistical parameters including central tendency (e.g. means) or other basic estimates (e.g. regression coefficient) AND variation (e.g. standard deviation) or associated estimates of uncertainty (e.g. confidence intervals)
☐	☒	For null hypothesis testing, the test statistic (e.g. *F*, *t*, *r*) with confidence intervals, effect sizes, degrees of freedom and *P* value noted<br>*Give P values as exact values whenever suitable.*
☒	☐	For Bayesian analysis, information on the choice of priors and Markov chain Monte Carlo settings
☐	☒	For hierarchical and complex designs, identification of the appropriate level for tests and full reporting of outcomes
☐	☒	Estimates of effect sizes (e.g. Cohen's *d*, Pearson's *r*), indicating how they were calculated

Our web collection on statistics for biologists contains articles on many of the points above.

Software and code

Policy information about availability of computer code

Data collection	No software was used for data collection
Data analysis	All analyses were conducted using R version 3.3.3 statistical software (R Foundation for Statistical Computing, Vienna, Austria).

For manuscripts utilizing custom algorithms or software that are central to the research but not yet described in published literature, software must be made available to editors/reviewers. We strongly encourage code deposition in a community repository (e.g. GitHub). See the Nature Research guidelines for submitting code & software for further information.

Data

Policy information about availability of data

All manuscripts must include a data availability statement. This statement should provide the following information, where applicable:

- Accession codes, unique identifiers, or web links for publicly available datasets
- A list of figures that have associated raw data
- A description of any restrictions on data availability

The data that support the findings of this study are available from Gallup but restrictions apply to the availability of these data, which were used under license for the current study. The data are not publicly available. Data are however available from the corresponding authors upon reasonable request and with permission of Gallup. A reporting summary for this Article is available as a Supplementary Information file. The source data underlying Figures 1-4 and Supplementary Figures 1-5 are provided as a Source Data file.

Field-specific reporting

Please select the one below that is the best fit for your research. If you are not sure, read the appropriate sections before making your selection.

☐ Life sciences ☒ Behavioural & social sciences ☐ Ecological, evolutionary & environmental sciences

For a reference copy of the document with all sections, see nature.com/documents/nr-reporting-summary-flat.pdf

Behavioural & social sciences study design

All studies must disclose on these points even when the disclosure is negative.

Study description	The study is a serial cross-sectional quantitative study.
Research sample	The current research reports results based on secondary data analyses of the Gallup World Poll (GWP), a dataset created by the Gallup Organization. The GWP includes nationally-representative samples (with some exceptions) from 163 countries (including Syria), representing 99% of the world's adult (15 or above) population. For example, in 2010, the Syrian sample included 49.0% women. The average age of the sample is 34.0. The study sample was chosen because it includes a large sample of over 11,000 Syrian participants from before to during the on-going Syrian Conflict.
Sampling strategy	Reference #24 provides a detailed description of the sampling strategy. The Gallup World Poll includes a mix of face-to-face interviews and telephone interviews. Multistage stratified random sampling was used in face-to-face interviews (e.g., in the Syrian sample). The country was first stratified into smaller geographical units, and the probability of participant selection was proportional to the population living in these units. This step ensures that the resulting sample provides good geographical coverage of the country. Then households were selected based on random route procedures. Once a household has been identified, a Kish grid is used to randomly select a participant from the household for the interview. In countries where telephone interview was used, random-digit-dialing was used. In both interview modes, multiple attempts were made to recruit participants to improve response rate. Survey weights were calculated by the GWP to ensure the representativeness of the sample.<br><br>The sample size in the Gallup World Poll was not determined by the current research team. The GWP aims to recruit at least 1,000 participants from each country for each annual survey. The rationale for using the GWP is that the GWP is the only dataset, to our knowledge, that includes well-being data from a large sample of 11,452 Syrians from before to during the Syrian Conflict. The sample size of the current study is much larger compared to past research on the Syrian conflict.<br><br>The following website provides the detailed description of the methodology of the GWP:<br>https://data-services.hosting.nyu.edu/wp-content/uploads/2017/10/World_Poll_Methodology_102717.pdf
Data collection	Reference #24 provides a detailed description of the data collection strategy. Gallup provided extensive training to local fieldworkers to ensure 1) the quality of the interview and 2) the random sampling procedure. Each interviewer had at least 1 full interview conducted in the presence of a fieldwork supervisor during first two days of data collection. A minimum of 5% of subsequent interviews were completed in the presence of a supervisor. We had no contact with the data collection team in Syria. Therefore, they were not aware of the hypotheses of the current manuscript during data collection.
Timing	Gallup Organization provides a detailed description of the timing of the data collection (cited as Reference 25). Reviewers and readers can refer to the document available in the following website:<br>https://www.gallup.com/file/services/177797/World_Poll_Dataset_Details__022318.pdf
Data exclusions	No data were excluded.
Non-participation	The Gallup World Poll uses a serial cross-sectional design. That is, the same participants were not followed over time. Therefore, attrition rate is not applicable here. Gallup Organization does not appear to share publicly the exact response rate for the GWP. However, when the Gallup Organization conducted a survey in Syria in 2009, they achieved an adequate response rate of 59% (Reference 27).
Randomization	No randomization was used. We controlled for sex and age in most analyses. The manuscript focused on examining and describing the longitudinal changes in physical, mental, and social well-being.

Reporting for specific materials, systems and methods

We require information from authors about some types of materials, experimental systems and methods used in many studies. Here, indicate whether each material, system or method listed is relevant to your study. If you are not sure if a list item applies to your research, read the appropriate section before selecting a response.

Materials & experimental systems

n/a	Involved in the study
☒	☐ Antibodies
☒	☐ Eukaryotic cell lines
☒	☐ Palaeontology
☒	☐ Animals and other organisms
☒	☐ Human research participants
☒	☐ Clinical data

Methods

n/a	Involved in the study
☒	☐ ChIP-seq
☒	☐ Flow cytometry
☒	☐ MRI-based neuroimaging
